# Activation of anti-inflammatory pathways by polyunsaturated fatty acid signaling may protect neurodevelopment in children prenatally exposed to methylmercury

**DOI:** 10.1186/s12940-026-01270-2

**Published:** 2026-04-16

**Authors:** Clara Bartra, Sabrina Llop, Julia Kuligowski, Abel Albiach-Delgado, Cristina Suñol, Eduard Rodríguez-Farré, Ferran Ballester, Raquel Soler-Blasco, Beatrice Jora, Júlia Jarné-Ferrer, Christian Griñán-Ferré, Santiago Vázquez, Mercè Pallàs, Coral Sanfeliu

**Affiliations:** 1 Department of Neurociències i Terapèutica Experimental, Institut d’Investigacions Biomèdiques de Barcelona (IIBB), CSIC, Barcelona, Spain; 2 PhD Program in Biotechnology, Facultat de Farmàcia i Ciències de l’Alimentació, Universitat de Barcelona, Barcelona, Spain; 3 Epidemiology and Environmental Health Joint Research Unit, FISABIO-UJI-UV, Valencia, Spain; 4 CIBERESP, Instituto de Salud Carlos III, Madrid, Spain; 5 Neonatal Research Group, Health Research Institute La Fe, Valencia, Spain; 6 Department of Nursing, Universitat de València, Valencia, Spain; 7 Laboratori de Química Farmacèutica, Facultat de Farmàcia i Ciències de l’Alimentació, Universitat de Barcelona, Barcelona, Spain; 8 Department of Pharmacology and Therapeutic Chemistry, Faculty of Pharmacy and Food Sciences, UBNeuro, Universitat de Barcelona, Barcelona, Spain; 9 CIBERNED, Instituto de Salud Carlos III, Madrid, Spain; 10 Institute of Biomedicine of the University of Barcelona (IBUB), Universitat de Barcelona, Barcelona, Spain

**Keywords:** Oxylipins, Polyunsaturated fatty acids, Methylmercury, Human perinatal samples, Fetal neuroprotection, Postnatal child neurodevelopment, 5XFAD mice, Soluble epoxide hydrolase, Epoxyeicosatrienoic acids.

## Abstract

**Background:**

Mercury is ubiquitous in the environment. Substantial levels of its organic form, methylmercury, pose a risk to fetal neurodevelopment through the maternal diet. Conversely, nutrients such as the polyunsaturated fatty acids (PUFAs) eicosapentaenoic acid (EPA), docosahexaenoic acid (DHA) and arachidonic acid (AA) promote neurodevelopment. We aimed to elucidate the neuroprotective role of PUFA signaling against prenatal methylmercury exposure in a Mediterranean fish-consuming area, addressing a critical gap in understanding nutritional modulation of neurotoxicity.

**Methods:**

Associations between levels of oxidized PUFA metabolites (oxylipins) in placental tissue (*N* = 12) or cord blood plasma (*N* = 39) and postnatal neurodevelopment in children were evaluated at two levels of prenatal mercury exposure. Low and moderate exposure groups were defined by total mercury concentrations in whole cord blood of 1.4–6.6 µg/L and 20–66 µg/L, respectively. Oxylipins were measured using liquid chromatography ­ mass spectrometry. Neurodevelopment was assessed at 14 months and 5 years using Bayley and McCarthy scales, respectively. Oxylipins were also analyzed in mouse brain tissue after treatment with a soluble epoxide hydrolase inhibitor to increase AA epoxide levels.

**Results:**

Under low mercury exposure, EPA-, DHA- and AA-derived placental oxylipins showed positive Spearman correlations with 14-month scores. In cord blood plasma, AA epoxides correlated positively with cognitive parameters at both ages under moderate exposure. Multivariate linear regression revealed positive associations between anti-inflammatory AA-derived epoxides and neurodevelopmental scores across tissues and ages, and between a DHA oxylipin in cord blood plasma and 14-month scores. No neurodevelopmental delays were observed in the moderate exposure group compared to the low exposure group. Oxylipin levels exhibited a more anti-inflammatory profile in samples from the moderate exposure group. Additionally, the brain oxylipin profile of mice treated with an AA epoxide enhancer indicated reduced inflammation.

**Conclusions:**

PUFA signaling dynamics revealed potential protective pathways against methylmercury-induced neurodevelopmental toxicity. Differential oxylipin modulation, together with the absence of neurodevelopmental delays, underscores the importance of a maternal diet rich in anti-inflammatory PUFAs for children at risk of cognitive impairment due to prenatal methylmercury exposure.

**Supplementary Information:**

The online version contains supplementary material available at 10.1186/s12940-026-01270-2.

## Background

Environmental exposure to methylmercury (MeHg), the most prevalent organic form of mercury, is a risk for human fetal neurodevelopment in areas with high mercury contamination. Mercury is a naturally occurring metal that is released into the atmosphere through natural emission sources and human industrial activities [1]. Near 5% of the inorganic mercury forms deposited in fresh water and seawater are converted to MeHg through microbial methylation [2]. This organic form then enters the food chain and accumulates in large, long-lived predatory fish. Fish consumption is the primary route of human exposure to MeHg [1]. However, MeHg can also be ingested through contaminated crops, particularly rice [3]. Two known poisonings episodes -those in Minamata, Japan in 1956 [4], and Iraq in 1972 [5]-, caused severe neurodevelopmental impairment in children born in the affected areas. Experimental studies have characterized MeHg as highly neurotoxic [6–8] and capable of crossing both the blood-placental barrier [9] and the blood-brain barrier [10]. At the molecular level, MeHg was initially recognized to bind to the thiol groups present in many peptides and proteins essential to cellular functions. More recently it has become clear that mercury also binds selenium with high affinity, irreversibly inhibiting selenoenzymes involved in protection against oxidative damage among other functions. Cellular depletion of biologically active selenium is now considered a primary driver of MeHg neurotoxicity [11–14]. Selenium contributes to mercury detoxification, but therapeutic options remain limited [15].

Therefore, there is a global effort to mitigate mercury emissions and reduce human exposure to MeHg [16–18]. The U.S. Environmental Protection Agency (US-EPA) has established a Reference Dose (RfD) of 0.1 µg/kg body weight/day for the lifelong safe dietary intake of MeHg [19]. The corresponding safety level in blood is 5.8 µg/L. This value was derived by applying a 10-fold uncertainty factor to the recommendation made in 2000 by a National Research Council (NRC, USA) committee, which identified 58 µg/L of MeHg in cord blood as a benchmark based on epidemiological studies of mother-infant pairs [20]. It is recommended that pregnant women avoid fish species that bioaccumulate MeHg [21]. However, MeHg intake easily surpasses the US-EPA RfD in various populations worldwide with high fish consumption [22]. Nevertheless, the risk posed by moderately elevated MeHg intake on fetal neurodevelopment remains controversial, with outcomes varying across populations and likely depending on maternal nutrient status [23–29].

Interestingly, an interaction between mercury exposure and fish nutrients had been reported in children’s neuropsychological development [30]. Modulation of the neurotoxic risk of MeHg by concurrent dietary selenium levels has been proposed as a plausible explanation for the differential population responses [11, 13]. Selenium is an essential micronutrient present in fish, which undoubtedly contributes to the protection of brain development. However, its benefits appear to depend on a narrow concentration range, and the relationship between prenatal selenium concentration and child neurodevelopment has been shown to follow an inverted U shape [31, 32]. In neurodevelopment, special attention has been paid to polyunsaturated fatty acids (PUFAs). Fish are rich in polyunsaturated fatty acids (PUFAs) that are transferred from maternal to fetal circulation together with MeHg [33]. Specifically, the long-chain PUFA eicosapentaenoic acid (EPA, C20:5 ω-3), docosahexaenoic acid (DHA, C22:4 ω-3), and arachidonic acid (AA, C20:4, ω-6), are involved in building the structure and functioning of the central nervous system [34].

Oxylipins, oxidized metabolites of PUFAs, are formed locally on demand via cytochrome P450 (CYP), cyclooxygenase (COX), and lipoxygenase (LOX) family enzymes. Free oxylipins interact with cell receptors or intracellular effectors to mediate their biological effects [35]. The COX and LOX pathways produce oxylipins that primarily affect inflammatory and vascular mechanisms, with either adverse or beneficial effects depending on their precursor fatty acid: AA or EPA, respectively [34]. LOX metabolism of DHA also produces oxylipins with generally anti-inflammatory and beneficial vascular effects [35]. CYP ω-hydroxylation results in a series of EPA metabolites with resolution of inflammation properties. Conversely, CYP epoxygenase products from linoleic acid (LA) have pro-inflammatory and generally adverse effects. However, the epoxy metabolites from EPA, DHA and AA are considered beneficial oxylipins. Specifically, AA-derived epoxyeicosatrienoic acids (EETs) are potent anti-inflammatory and neuroprotective agents [36, 37] and may protect the developing brain against genetic or environmental insults that would otherwise cause cognitive impairment, as shown in experimental studies [38, 39].

We aimed to investigate the influence of oxylipins levels in placental tissue and cord blood of children prenatally exposed to two different levels of mercury on postnatal neurocognitive and psychomotor development at 14 months and 5 years of age. We hypothesized that this would reveal the activation of PUFA signaling pathways involved in counteracting potential MeHg-induced stress. Co-exposure with cadmium and lead, two other widespread developmental neurotoxic agents, was analyzed in selected settings. To further explore AA signaling, we also aimed to identify changes in the brain oxylipin profile of wild-type (WT) mice and 5XFAD mice -a transgenic Alzheimer’s disease model (TgAD)- following a treatment that increases EETs.

## Materials and methods

### Human samples of placenta and cord blood

The use of human samples and all the procedures of the study were approved by the *Comité Coordinador de Ética de la Investigación Biomédica de Andalucía *(approval codes S1900596 and 2218-N-19, from date 07/31/2020), and the Ethics Committees of Hospital La Fe de Valencia (approval date: 10/27/2004) and the CSIC (approval code: 072/2019). Informed consent was obtained from all participants, and their privacy rights were protected. The study adhered to the principles of the Declaration of Helsinki. Clinical trial number: not applicable.

Tissue samples of placenta (pairs of fetal and maternal parts) and cord blood plasma of children from the INMA (Childhood and Environment Project) cohort in the Valencia region (Spain) were obtained from the *Biobanco del Sistema Sanitario Público de Andalucía* and Fisabio Biobank, respectively. The two tissues were from independent donors. This cohort is part of the multicenter INMA Spanish Study on the effects of prenatal environmental exposures on child health [33, 40]. Whole cord blood from all donors had previously been analyzed for total mercury (T-Hg), a reliable biomarker of fetal MeHg exposure [41, 42]. The T-Hg analytical procedure is described elsewhere [33]. Maternal levels of cadmium and lead have been previously measured from urine samples during pregnancy [43]. The cohort is from a Mediterranean coastal area with high fish consumption, and many children had cord blood T-Hg levels exceeding the US-EPA RfD for MeHg intake, similar to other fish-eating populations [30, 33]. We selected cohort donors with the lowest and highest T-Hg levels to highlight potential differences associated with varying prenatal mercury exposure. Exposure levels for each tissue donor were arbitrarily categorized as “low” (T-Hg: 1.4 to 6.6 µg/L) or “moderate” (T-Hg: 20 to 66 µg/L). Sample details for sex and T-Hg levels are listed in Supplementary Table 1. The demographic profile of tissue donors and T-Hg values per group are shown in Table 1.

**Table 1:** Characteristics of the participating women and their newborns in the study groups of the INMA Project, Valencia

	Pairs of fetal and maternal placenta donors		Cord blood plasma donors	
	Low T-Hg	Moderate T-Hg	Low T-Hg	Moderate T-Hg
T-Hg (µg/L)	4.3 (2.7–5.9)	23.5 (20.0-27.5)*	1.4 (1.4-3.0)	42.0 (40.0–51.0)**
Number	6	6	20	19
Maternal variables				
Age (y)	30.0 (24.2–39.2)	30.5 (26.0–36.0)	26.0 (23.0–30.0)	33.0 (30.0–35.0)**
Country of birth				
Spain	4 (66.7)	6 (100)	14 (70)	19 (100)
Other	2 (33.3)	0 (0)	6 (30)	0 (0)
Place of residence				
Rural	0 (0)	0 (0)	1 (5)	1 (5.3)
Suburban	1 (16.7)	0 (0)	0 (0)	2 (10.5)
Urban	5 (83.3)	6 (100)	19 (95)	16 (84.2)
Education				
Up to primary	5 (83.3)	3 (50)	8 (40)	7 (36.8)
Secondary	1 (16.7)	2 (33.3)	9 (45)	9 (47.4)
University	0 (0)	1 (16.7)	3 (15)	3 (15.8)
Social class				
I + II	0 (0)	1 (16.7)	0 (0)	2 (10.5)
III	3 (50)	1 (16.7)	8 (40)	2 (10.5)
IV + V	3 (50)	4 (66.6)	12 (60)	15 (78.9)
Pre-pregnancy BMI (kg/m^2^)	24.2 (20.4–28.2)	22.1 (20.5–24.8)	24.0 (19.7–27.9)	23.5 (19.7–29.0)
Seafood consumption (g/week)	65.3 (48.0–104.6)	62.8 (50.2–74.1)	56.6 (27.0-68.7)	102.1 (69.6-123.8)*
Employment during pregnancy				
No	1 (16.7)	0 (0)	3 (15.8)	6 (31.6)
Yes	5 (83.3)	6 (100)	16 (84.2)	13 (68.4)
Maternal smoking during pregnancy				
No	1 (16.7)	1 (16.7)	9 (47.4)	6 (31.6)
Yes	5 (83.3)	5 (83.3)	10 (52.6)	13 (68.4)
Urinary cadmium (Cd) during pregnancy (µg/g creatinine)	N.D.	N.D.	0.16 (0.13–0.51)	0.17 (0.12–0.43)
Urinary lead (Pb) during pregnancy (µg/g creatinine)	N.D.	N.D.	1.06 (0.56–2.01)	1.09 (0.73–2.64)
Newborn variables				
Preterm gestation (< 37 weeks)				
No	6 (100)	6 (100)	20 (100)	17 (89.5)
Yes	0 (0)	0 (0)	0 (0)	2 (10.5)
Low weight at birth (< 2500 g)				
No	6 (100)	6 (100)	19 (95)	19 (100)
Yes	0 (0)	0 (0)	1 (5)	0 (0)
Breastfeeding (weeks)	15.9 (0.0-38.1)	18.3 (3.3–39.1)	21.6 (1.1–30.3)	11.8 (0.0-40.3)
Number of siblings				
0	2 (33.3)	4 (66.7)	10 (50.0)	8 (42.1)
1	2 (33.3)	2 (33.3)	6 (30.0)	9 (47.4)
≥ 2	2 (33.4)	0 (0)	4 (20.0)	2 (10.5)
Newborn sex				
Male	4 (66.7)	3 (50)	10 (50)	14 (73.7)
Female	2 (33.3)	3 (50)	10 (50)	5 (26.3)

Data are shown as median (IQR) or number (%). N.D., not determined. Statistics: Mann Whitney test or Chi square test, * *p* < 0.01, ** *p* < 0.001 compared to low T-Hg group. T-Hg, total mercury in whole cord blood

### Neuropsychological assessment of children

Child neurodevelopment has previously been evaluated through neuropsychological tests conducted as part of the INMA Project [44]. Here we present the results for the children selected for this study. Specifically, cognitive and psychomotor development was comprehensively assessed at around 14 months of age using the *Bayley Scales of Infant and Toddler Development III* (BSID) [45], and at around 5 years of age using the *McCarthy Scales of Children’s Abilities* (MSCA) [46]. The latter was a standardized version adapted for the Spanish population. The tests used were validated for the respective ages assessed. For BSID and MSCA scales, composite scores of 100 (standard deviation 15 and 16, respectively) indicate normal neurodevelopment. Procedures were carried out as described elsewhere [30, 47]. In brief, the BSID includes the Mental scale (163 items) and the Psychomotor scale (81 items). The MSCA comprises 18 subtests that yield test scores across cognitive and motor domains, including verbal information processing, perceptual performance, numerical abilities, short-term memory, and gross and fine motor skills. Additional scores for executive function, working memory and general cognition were also calculated. All neuropsychological data were adjusted for children’s age.

### Brain tissue of mice treated with a soluble epoxide hydrolase inhibitor 

Inhibition of the soluble epoxide hydrolase enzyme (sEH) curtails the hydrolysis of AA-derived EETs into their less active diol metabolites, dihydroxieicosatrienoic acids (DiHETs), thereby increasing the anti-inflammatory activity of EETs. Subsequent shifts in the balance of inflammation-related oxylipins were investigated in mouse brain.

The use of mouse tissue samples and animal procedures were approved by the local *Comitè Ètic d’Experimentació Animal*,* Universitat de Barcelona* (CEEA-UB) following the guidelines of the Animal Experimentation Commission of the Autonomous Government of Catalonia (approval code #10291, 1/28/2018). All procedures were conducted in accordance with the European Commission Council Directive 86/609/EEC of 24 November 1986 and associated guidelines.

A novel sEH inhibitor (sEHi) was used in this study. UB-BJ-02, 1-(9-Fluoro-5,6,8,9,10,11-hexahydro-7*H*-5,9:7,11-dimethanobenzo[9]annulen-7-yl)-3-(1-(tetrahydro-2*H*-pyran-4-carbonyl)piperidin-4-yl)urea, was synthesized from 9-fluoro-5,6,8,9,10,11-hexahydro-7*H*-5,9:7,11-dimethanobenzo[9]annulen-7-amine hydrochloride and (4-aminopiperidin-1-yl) (tetrahydro-2*H*-pyran-4-yl)methanone following a previously reported procedure [48].

Cortical brain tissue samples were collected from female and male WT and 5XFAD mice treated with the UB-BJ-02 at a dose of 5 mg/Kg or with vehicle (*N* = 5 per group). The compound was administered via drinking water for 4 weeks. Control animals received water containing 1.8% (2-hydroxypropyl)-β-cyclodextrin (vehicle). Mice were 6 month old at the time of termination. The UB-BJ-02 treatment has been reported to decrease neuroinflammation markers and memory loss in 5XFAD mice [49].

### Molecular markers of placental tissue

The presence of deleterious effects of mercury in the moderate T-Hg group was assessed in the placental tissues. For each placenta, results were calculated as the average of the corresponding pair of fetal and maternal placental tissue samples.

Enzymatic activity of the cell proteasome system was analyzed using the Proteasome-Glo™ Assay Systems (Promega, USA). Specific luminogenic substrates were added to the supernatants of placenta lysates to measure chymotrypsin-like, trypsin-like, and caspase-like activities. Assays were performed as described elsewhere [50].

Epigenetic changes of histone acetylation and methylation were analyzed using ELISA-based colorimetric methods. Tissue homogenates were assayed for global acetylation of histone H3 and H4 using the EpiQuik™ Global Histone H3 Acetylation Assay Kit and EpiQuik™ Global Histone H4 Acetylation Assay Kit (EpigenTek, Farmingdale, NY, USA), respectively. Histone methylation changes were assayed by extracting histones from tissue samples and quantifying two key methylation sites using the EpiQuik™ Total Histone Extraction Kit, EpiQuik™ Global Tri-Methyl Histone H3K27 Quantification Kit, and EpiQuik Global Tri-Methyl Histone H4K20 Quantification Kit (EpigenTek), respectively. All procedures were performed according to the manufacturer’s instructions. Final optical density measurements were recorded in duplicate wells using a plate spectrometer at 450 nm (Multiskan Sky, Thermo Fisher Scientific).

### Oxylipins analysis

The oxylipin profile of all tissue samples was determined using the Acquity-Xevo TQ-XS ultraperformance liquid chromatography coupled to tandem mass spectrometry (UPLC-MS/MS) system from Waters (Milford, MA, USA), operating in negative electrospray ionization mode (ESI-), and using a Waters Acquity UPLC BEH C18 columns (2.1 × 100 mm, 1.7 μm). This methodology allows the accurate determination of a wide panel of oxylipins [51]. Standards, reagents, and the detailed procedure are described in the Supplementary Material. In brief, chemicals were of the highest available purity. Approximately 20 mg of placental and cortical tissue samples were homogenized in 0.1% ammonium acetate. Then, 200 µL of supernatant from tissues or plasma samples were spiked with internal standards and 3,5-di-*tert*-4-butylhydroxytoluene. Extraction and preconcentration of oxylipins were performed after the addition of 200 µL of methanol (0.1% formic acid), centrifugation, and dilution with 1400 µL H_2_O, followed by a solid-phase extraction (SPE) procedure with Oasis-HLB 96-well plates (30 mg) from Waters. Final extracts were evaporated and redissolved in 50 µL of MeOH: CH_3_CN (50:50)% (v/v) and analyzed by UPLC-MS/MS. For data analysis, raw chromatograms were processed using MassLynx v4.2 software from Waters, including peak area integration and interpolation in external calibration lines. The method enabled the simultaneous determination of 32 oxygenated PUFA metabolites listed in Supplementary Table 2. However, some oxylipins could not be determined because their concentrations were below the limits of detection (LOD), which were 0.7 nmol/mg of tissue in placenta and mouse brain samples and 0.04 nM in plasma samples. Limits of quantification (LOQ) were 2.2 nmol/mg and 0.13 nM for tissue and plasma samples, respectively. In rare cases where oxylipins were detected but not quantifiable, a value of LOQ x 0.5 was assigned. Oxylipins included in the study are described in Supplementary Table 3. Results for each pair of fetal and maternal placental tissues were averaged after separate analysis.

### Statistics

Placental and umbilical cord blood samples were analyzed separately due to their different donor origin. Demographic data were described according to T-Hg levels (low and moderate), using median and interquartile range (IQR) for continuous variables, and frequencies and percentages for categorical variables. Group differences were assessed using the Mann–Whitney U test for continuous variables and the Chi-square test for categorical variables. Power analysis indicated that the pooled plasma sample size provided good statistical power, while the segregated exposure group design had moderate power and might not detect minor effects. The small placenta group sizes resulted in lower statistical power but allowed detection of large effects and moderate to strong associations. For the mouse study, sample size calculation for four experimental groups using the resource equation method yielded an appropriate size. Associations between oxylipin levels and neurodevelopmental scores were first explored using Spearman’s nonparametric bivariate correlation analysis. Spearman correlations were calculated within each group of low or moderate prenatal mercury exposure and for pooled data, for each tissue. Results are shown as Spearman ρ coefficients and their statistical significances. Correlation strength is considered moderate for ρ values between 0.40 and 0.59, strong for ρ between 0.60 and 0.79, and very strong for ρ values between 0.80 and 1.00. Next, a multivariate linear regression analysis was performed using pooled data from the two T-Hg exposure groups. The potential covariates were selected from previous results in this cohort [25, 47] (maternal age, body mass index (BMI), maternal education, breastfeeding and newborn sex). Following a backward elimination procedure, variables associated in a multiple model with a *p*-value < 0.10 in the likelihood ratio test (LRT) were retained in the models. Sex of the newborn and cord blood T-Hg levels were forced in the models independently of their statistical significance. Maternal seafood consumption was also forced as possible confounder. Results are shown as ß coefficients, 95% confidence intervals (CI) and their statistical significances for the relationship between oxylipin levels and neurodevelopmental scores. All other results are shown as mean ± standard error of the mean (SEM) for each group. Normality was assessed using the Shapiro–Wilk test or the Kolmogorov-Smirnov test. Significant differences between two groups were analyzed using the two-tailed Student’s *t* test. ANOVA followed by Tuckey’s post hoc test was applied for designs with multiple groups. Statistics was performed using IBM SPSS v23 software (IBM Corp., Armonk, NY, USA) and GraphPad Prism v6.01 (GraphPad Software, La Jolla, CA, USA). Heat maps were generated using Microsoft Excel v16.77.1 (Microsoft Corporation, Redmond, WA, USA).

## Results

### Molecular changes in placental tissues following differential prenatal mercury exposure

**Fig. 1:**
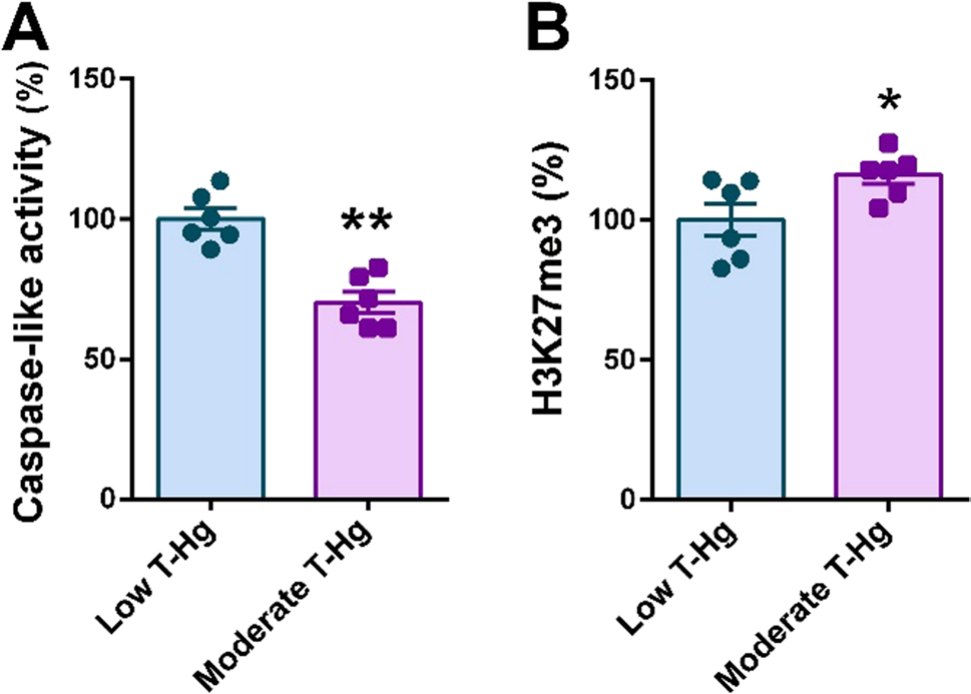
Proteasome function and epigenetic modifications in placental tissues across two prenatal mercury exposure groups. **A** Caspase-like enzymatic activity of the proteasome (*N* = 6 per group). **B** Levels of trimethylation of lysine 27 on histone H3 (H3K27me3) (*N* = 6 per group). Statistics: Student’s *t* test, * *p* < 0.05, ** *p* < 0.01. T-Hg, total mercury in whole cord blood

The analysis of the placental tissues showed mild deleterious effects on both proteasome and epigenetic biomarkers in the moderate mercury exposure group of this cohort (Fig. 1). Proteasome enzymatic activities were moderately impaired, with a 30% reduction observed in caspase-like activity (Fig. 1A), while no significant changes were detected in trypsin-like or chymotrypsin-like activities (Supplementary Fig. 1A and 1B, respectively), compared to the low mercury exposure group. Regarding epigenetic histone modifications, samples from donors with moderate mercury exposure showed a significant 16% increase in H3 trimethylation at lysine 27 (H3K27me3), a mark associated with transcriptional repression (Fig. 1B). However, no significant changes were observed in the other histone epigenetic markers analyzed (Supplementary Fig. 1C-E).

### Changes in oxylipin profiles of placental tissue and cord blood plasma following differential prenatal mercury exposure

**Fig. 2:**
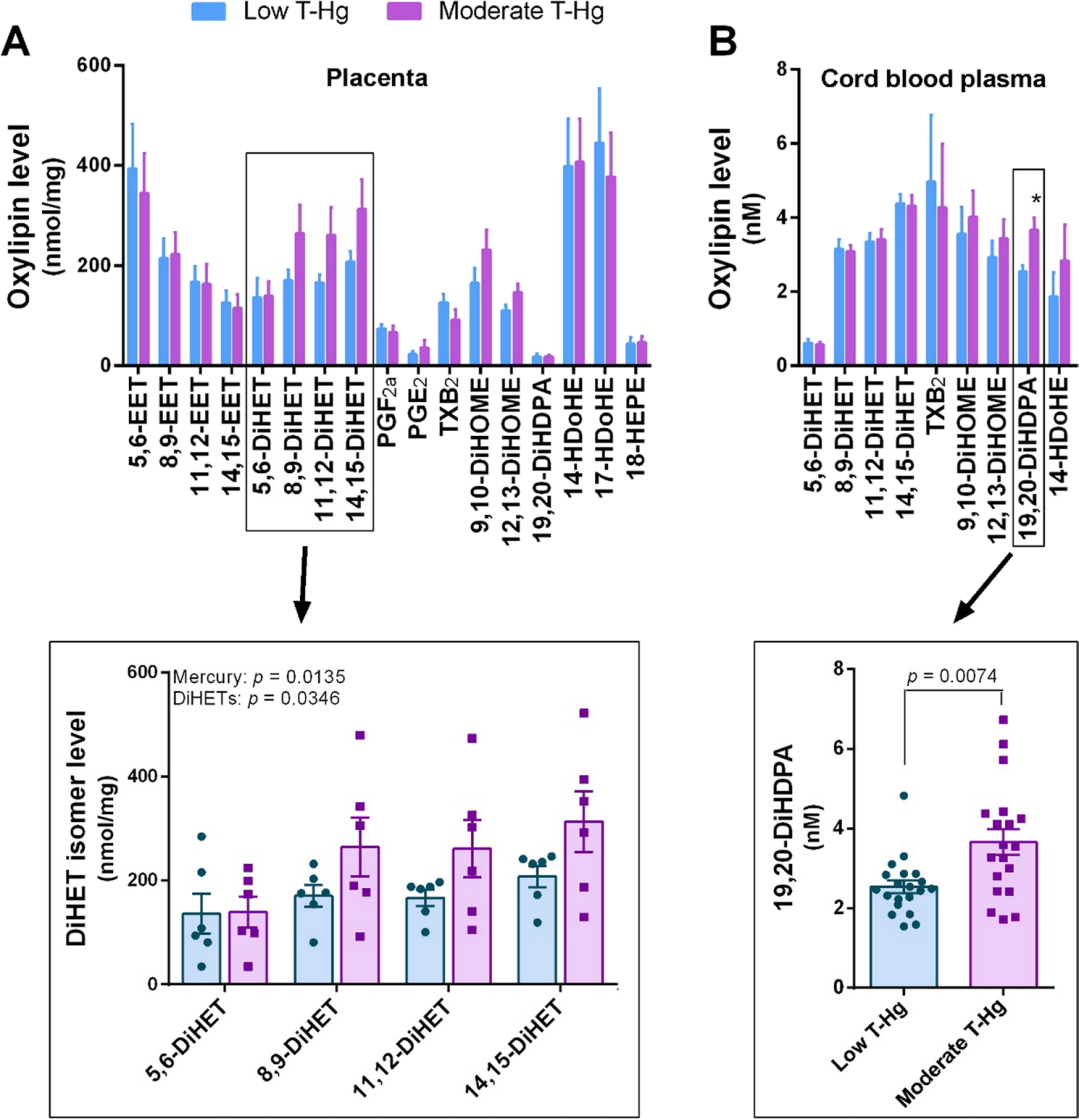
Oxylipin profiles in placental tissue and cord blood plasma across two prenatal mercury exposure groups. **A** Levels of 17 oxylipins detected in placental tissue (*N* = 6 per group); the boxed graph below shows levels of DiHET isomers with two-way ANOVA statistics. **B** Levels of 9 oxylipins detected in cord blood plasma (*N* = 19–20 per group); the boxed graph below shows levels of 19,20-DiHDPA analyzed using Student’s *t* -test. Statistics: * *p* < 0.01; significance of ANOVA factors is indicated in the graph. T-Hg, total mercury in whole cord blood

UPLC-MS/MS analysis of the placental tissues showed detectable levels of 17 oxylipins (Fig. 2A). Pairwise comparison between relatively low and moderate environmental mercury exposure did not yield statistically significant differences in oxylipin levels. However, there was a trend toward a mercury-induced increase in three of the four DiHET isomers of the CYP-epoxygenase AA pathway. Two-way ANOVA of DiHETs levels indicated significant effects of both mercury exposure and isomer type (bottom boxed graph in Fig. 2A). These metabolites are produced via hydrolysis of the corresponding EET isomers.

Analysis of cord blood plasma samples showed detectable levels of 9 oxylipins (Fig. 2B). Among these, the CYP epoxygenase metabolite from DHA, (±)19,20-dihydroxy-4Z,7Z,10Z,13Z,16Z-docosapentaenoic acid (19,20-DiHDPA), exhibited increased levels in the moderate mercury exposure group (bottom boxed graph in Fig. 2B).

### Neurodevelopment of children was not impaired by prenatal mercury exposure

Neuropsychological assessment of the children included in the study did not reveal any significant differences associated with prenatal mercury exposure at 14 months and 5 years of age. All children scored within the expected average range for normal neurodevelopment.

**Fig. 3:**
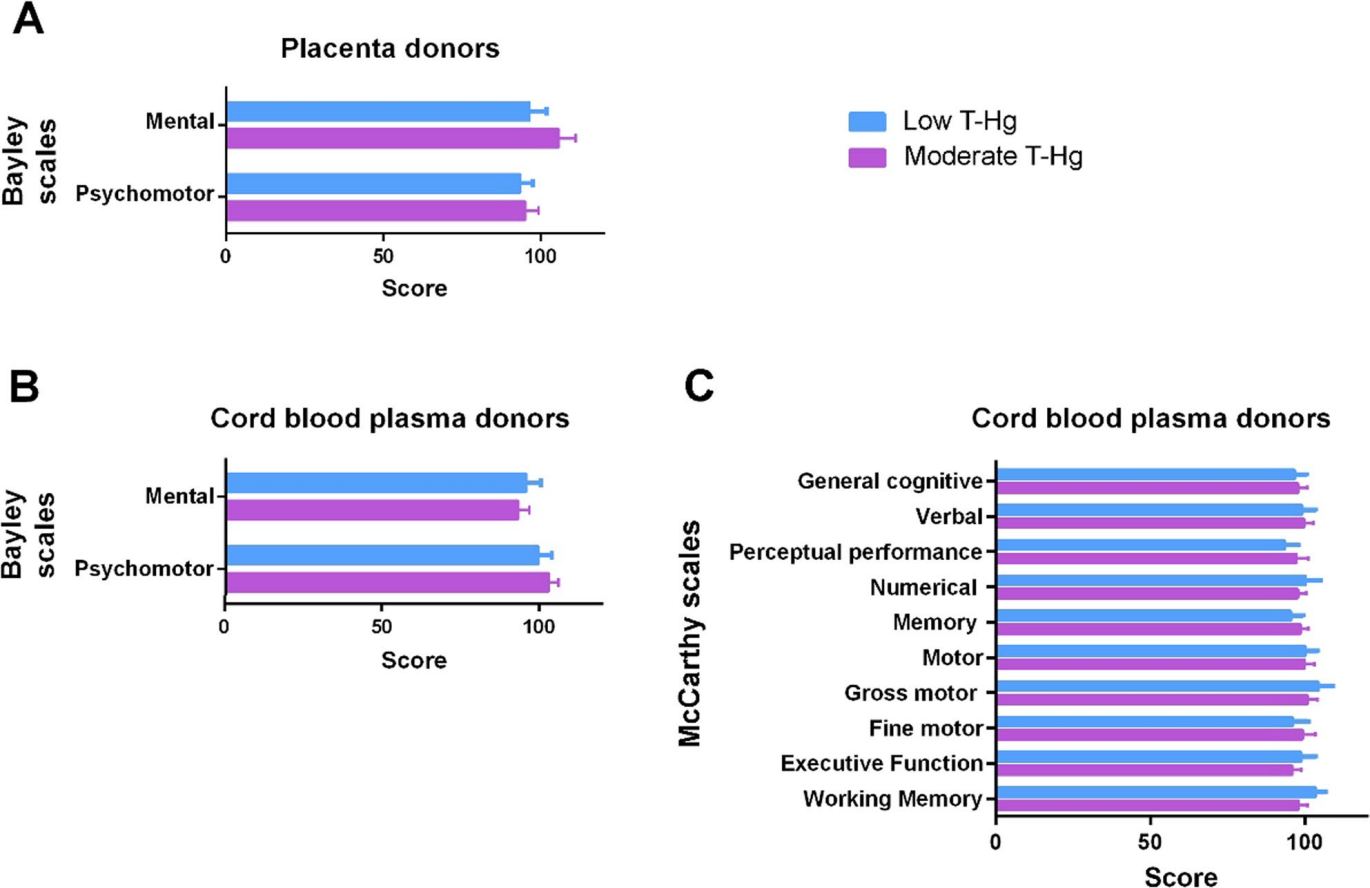
Neurodevelopmental scores of children at 14 months and 5 years of age across two prenatal mercury exposure groups. **A** Bayley Scales of Infant and Toddler Development at 14 months of age in placental tissue donors (*N* = 6 per group). **B** Bayley Scales of Infant and Toddler Development in cord blood plasma donors (*N* = 16–18 per group). **C** McCarthy Scales of Children’s Abilities scores at 5 years of age in cord blood plasma donors (*N* = 8–17 per group). Statistics: No significant differences were observed between the low and moderate prenatal mercury exposure groups for any score. T-Hg, total mercury in whole cord blood

At 14 months of age, direct comparisons of Bayley scale scores between children prenatally exposed to relatively low and moderate mercury levels showed no statistical differences, either in the groups with placental samples (Fig. 3A) or in those with cord blood plasma samples (Fig. 3B).

Similarly, at 5 years of age, analysis of McCarthy scores in the cord blood plasma group showed no significant differences related to mercury exposure (Fig. 3C). McCarthy scores for the placenta donor group were not statistically evaluated due to the small sample size in both exposure groups (*N* = 3–4). However, the available data did not suggest any adverse effects of mercury exposure (Supplementary Table 4).

### Associations of oxylipin profile with neurodevelopmental scores following differential prenatal mercury exposure

#### Analysis of data categorized by low or moderate exposure to mercury at each age and tissue

**Fig. 4:**
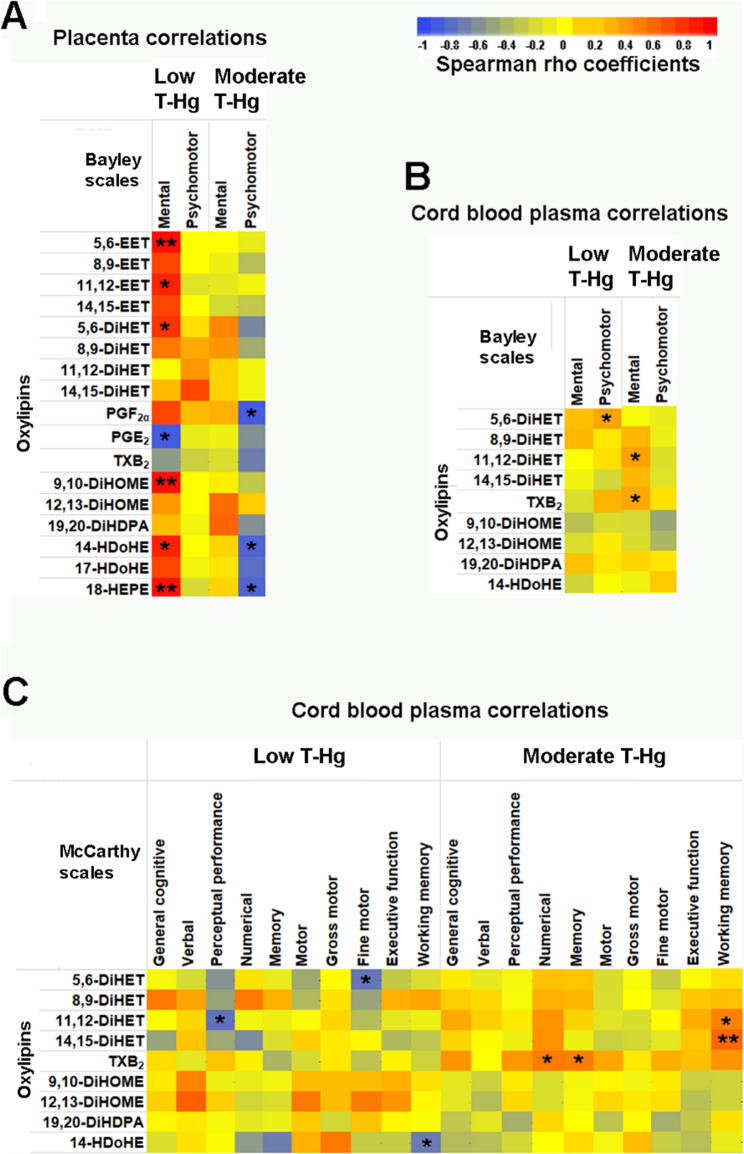
Spearman correlations between neurodevelopmental scores and oxylipin levels across two prenatal mercury exposure groups. Correlation strength is represented as a heat map of Spearman ρ coefficients. **A** Correlations between oxylipin levels in placental tissues and Bayley scale scores at 14 months of age for low and for moderate mercury group (*N* = 6 per group). **B** Correlations between oxylipin levels in cord blood plasma and Bayley scale scores for the low mercury group (*N* = 16) and moderate mercury group (*N* = 18). **C** Correlations between oxylipin levels in cord blood plasma and McCarthy scale scores at 5 years of age for the low mercury group (*N* = 8) and moderate mercury group (*N* = 17). Statistics: * *p* < 0.05, ** *p* < 0.01. The heat map scale indicates the values of Spearman’s rho coefficient for the three panels A, B and C. T-Hg, total mercury in whole cord blood

Associations of oxylipin levels with postnatal neurodevelopmental scores within each group of relatively low and moderate mercury exposure were analyzed using Spearman correlations. At 14 months of age, several strong or very strong correlations were observed between the scores of Bayley scales and the levels of some oxylipins analyzed in pairs of fetal and maternal placental tissues within each mercury exposure group. Results are presented as a heat map of Spearman correlation coefficients (Fig. 4A), with corresponding numerical data shown in Supplementary Table 5. In the low mercury group, significant positive correlations were found between the Bayley mental scale and several EET and DiHET isomers, along with other anti-inflammatory oxylipins derived from DHA (14-hydroxydocosahexaenoic acid, 14-HDoHE) and EPA (18-hydroxyeicosapentaenoic acid, 18-HEPE). A negative correlation was observed between the mental scale and the AA-derived pro-inflammatory prostaglandin E_2_ (PGE_2_), along with an unexpected positive correlation with the toxic signaler 9,10-dihydroxy-12Z-octadecenoic acid (9,10-DiHOME), derived from LA. In the moderate mercury group, no positive correlations were observed. However, negative correlations were found between the Bayley psychomotor scale and prostaglandin F_2α_ (PGF_2α_), as well as the anti-inflammatory oxylipins 14-HDoHE and 18-HEPE.

Oxylipin levels in cord blood plasma showed some moderate correlations with Bayley scale scores, as illustrated in the corresponding heat map (Fig. 4B), with numerical data in Supplementary Table 6. Several DiHET isomers positively correlated with Bayley scales, including two significant correlations: one with the psychomotor scale in the low mercury exposure group, and another with the mental scale in the moderate exposure group. The AA-derived pro-inflammatory thromboxane B_2_ (TXB_2_) also showed a significant positive correlation with the mental scale in the moderate exposure group.

At 5 years of age, correlations between oxylipin levels in placental tissues and McCarthy scale scores could not be independently assessed for low and moderate mercury exposure groups due to the limited number of cases in each group.

McCarthy scales showed several strong correlations with oxylipins measured in cord blood plasma, as shown in the heat map (Fig. 4C), with numerical data in Supplementary Table 7. In the low mercury group, negative correlations were found between two DiHET isomers and perceptual performance and fine motor scores, respectively, as well as between the anti-inflammatory oxylipin 14-HDoHE and working memory. In contrast, the moderate mercury exposure group showed positive correlations between two DiHET isomers and working memory. Additionally, in this group, positive correlations were observed between the pro-inflammatory TXB_2_ and both numerical and memory scores.

We cannot rule out the possibility of false associations due to the reduced sample size and the corresponding limitations in statistical analysis. This may apply to some negative correlations observed between beneficial oxylipins and neurodevelopmental scores in placenta and cord blood at 14 months and 5 years of age, respectively.

#### Analysis of data grouped for each age and tissue

To gain further insight into the potential benefits of oxylipins, the association of their profile and postnatal neurodevelopmental scores was also analyzed in pooled data from both mercury exposure groups through a multivariate regression analysis.

The association of Bayley scales scores at 14 months with placental oxylipin level are shown in Table 2. All four EET isomers analyzed showed borderline significance (*p* < 0.1) in the mental scale with positive coefficients. These results are in agreement with moderate Spearman correlations for several EET and DiHET isomers, with statistical significance observed for 5,6-DiHET. Spearman correlations of the mental scores with the oxylipins 9,10-DiHOME, 14-HDoHE and 18-HEPE, which were previously found in the low-exposed T-Hg group, were maintained in the pooled samples. In addition, TXB_2_ showed a negative association with the psychomotor scale. No further associations were detected.

**Table 2:** Association between Bayley scales at 14 months of age and 17 oxylipins detected in fetal and maternal placental tissue pairs for all children (*N* = 12)

	BSID^a^ scores	Mental scale					Psychomotor scale				
		Multivariate linear regression			Spearman correlation		Multivariate linear regression			Spearman correlation	
		Beta	95% CI	*p*	Rho	*p*	Beta	95% CI	*p*	Rho	*p*
Oxylipins (nM)	5,6-EET	**0.038**	0.00; 0.08	0.052	0.455	0.137	0.003	-0.04; 0.05	0.890	0.077	0.812
	8,9-EET	**0.072**	-0.01; 0.15	0.066	0.483	0.111	0.007	-0.08; 0.09	0.850	0.007	0.983
	11,12-EET	**0.079**	-0.01; 0.17	0.077	0.385	0.216	0.004	-0.10; 0.10	0.924	-0.021	0.948
	14,15-EET	**0.117**	-0.01; 0.24	0.065	0.389	0.212	0.007	-0.14; 0.15	0.913	-0.133	0.681
	5,6-DiHET	0.126	-0.12; 0.37	0.262	0.619*	0.032	0.091	-0.13; 0.31	0.360	-0.147	0.648
	8,9-DiHET	0.016	-0.10; 0.13	0.752	**0.515**	0.087	0.042	-0.05; 0.14	0.326	0.056	0.863
	11,12-DiHET	0.003	-0.10; 0.11	0.946	0.305	0.335	0.044	-0.04; 0.13	0.258	0.371	0.235
	14,15-DiHET	0.008	-0.09; 0.10	0.849	0.406	0.190	0.046	-0.03; 0.12	0.175	0.413	0.183
	PGF_2α_	0.124	-0.46; 0.71	0.631	**0.504**	0.094	0.042	-0.48; 0.56	0.855	-0.343	0.276
	PGE_2_	-0.178	-0.53; 0.18	0.278	-0.399	0.198	-0.158	-0.47; 0.15	0.268	-0.385	0.217
	TXB_2_	-0.024	-0.24; 0.19	0.794	-0.329	0.296	-0.141*	-0.28; 0.00	0.047	-0.441	0.152
	9,10-DiHOME	0.034	-0.11; 0.18	0.587	0.613*	0.034	0.033	-0.09; 0.16	0.542	-0.063	0.846
	12,13-DiHOME	0.106	-0.18; 0.39	0.413	0.410	0.186	0.108	-0.14; 0.35	0.334	0.203	0.527
	19,20-DiHDPA	-0.095	-0.97; 0.78	0.804	0.378	0.225	0.013	-0.76; 0.78	0.970	-0.168	0.602
	14-HDoHE	0.039	-0.02; 0.10	0.189	0.606*	0.037	-0.005	-0.07; 0.06	0.866	-0.294	0.354
	17-HDoHE	0.033	-0.04; 0.10	0.304	**0.525**	0.079	0.021	-0.04; 0.08	0.467	-0.245	0.443
	18-HEPE	0.209	-0.16; 0.58	0.221	0.602*	0.038	-0.043	-0.40; 0.32	0.784	-0.350	0.265

The two statistical models were independently calculated with the same data. Multivariate linear regression model adjusted for cord blood T-Hg concentration, maternal seafood consumption, and newborn sex

^a^*BSID* Bayley Scales of Infant and Toddler Development

Highlighted significant values (* *p* < 0.05) and borderline values (*p* < 0.1)

McCarthy scores revealed scattered positive Spearman correlations with placental oxylipins, as shown in Supplementary Table 8. The analysis showed very strong positive correlations of 5,6-DiHET and 18-HEPE with motor and fine motor scores. Furthermore, positive correlations were observed between the two anti-inflammatory oxylipins, 17-HDoHE and 18-HEPE, and the fine motor score. The association between the oxylipin profile in placental tissue samples and neurodevelopmental scores at 5 years was not analyzed using multivariate linear regression due to the limited sample size.

Results of the association of Bayley scale scores at 14 months with plasma oxylipin profile in pooled samples are shown in Table 3. Positive association between several DiEHT isomers and the mental scores were observed. Multivariate linear regression model also revealed a significant positive association between 19,20-DiHDPA and the mental scale, which did not reach significance in the Spearman correlation analysis. Associations between the oxylipin profile and the psychomotor scale were detected in the Spearman analysis, showing positive correlations with 5,6-DiHET, 19,20-DiHDPA, and TXB_2_.

**Table 3:** Association between Bayley scales at 14 months of age and 9 oxylipins detected in cord blood plasma for all children (*N* = 34)

	BSID^a^ scores	Mental scale					Psychomotor scale				
		Multivariate linear regression			Spearman correlation		Multivariate linear regression			Spearman correlation	
		Beta	95% CI	*p*	Rho	*p*	Beta	95% CI	*p*	Rho	*p*
Oxylipins (nM)	5,6-DiHET	**18.35**	-1.38; 38.08	0.067	0.276	0.114	9.23	-9.21; 27.67	0.314	0.352*	0.041
	8,9-DiHET	11.40**	4.14; 18.64	0.003	0.437**	0.010	3.03	-4.36; 10.43	0.408	**0.288**	0.099
	11,12-DiHET	7.08**	1.91; 12.26	0.009	0.411*	0.016	1.70	-3.38; 6.78	0.498	0.188	0.287
	14,15-DiHET	4.54	-1.01; 10.09	0.105	**0.321**	0.064	-0.102	-5.20; 5.00	0.968	0.006	0.973
	TXB_2_	-0.218	-0.98; 0.54	0.563	0.254	0.147	0.003	-0.67; 0.67	0.992	0.386*	0.024
	9,10-DiHOME	-0.580	-3.06; 1.89	0.635	-0.084	0.638	-0.20	-2.48: 2.08	0.860	-0.164	0.353
	12,13-DiHOME	-0.54	-4.13; 3.06	0.763	-0.020	0.912	0.118	-3.08; 3.32	0.940	-0.003	0.986
	19,20-DiHDPA	6.48**	1.78; 11.18	0.009	**0.305**	0.080	3.66	-0.83; 8.14	0.106	0.365*	0.034
	14-HDoHE	-0.39	-2.03: 1.24	0.625	0.026	0.885	0.293	-1.14; 1.72	0.678	**0.333**	0.054

The two statistical models were independently calculated with the same data. Multivariate linear regression model adjusted for cord blood T-Hg concentration, maternal seafood consumption and newborn sex. Mental scale additionally adjusted for maternal age and psychomotor scale for maternal BMI

^a^*BSID* Bayley Scales of Infant and Toddler Development. Highlighted significant values (* *p* < 0.05, ** *p* < 0.01) and borderline values (*p* < 0.1)

The pooled sample analysis of associations between the McCarthy scales at 5 years of age and cord plasma oxylipins is shown in Table 4 and Supplementary Table 9 A and B. The multivariate linear regression models showed significant or borderline positive associations of various DiEHT isomers with the perceptual performance, numerical, and working memory scales (Table 4). A marginal negative association was observed with the fine motor scale. TXB_2_ was also associated with the perceptual performance scale. The Spearman correlation analysis of these scales showed similar results. Analysis of the general cognitive, gross motor, and executive function scales revealed scattered associations in the Spearman correlation model (Supplementary Table 9 A). Specifically, TXB_2_ and 14-HDoHE showed positive correlations, while 14-HDoHE also showed a negative correlation trend with these scales. Furthermore, the verbal, memory, and motor scales did not show any associations (Supplementary Table 9B). The previously analyzed Spearman correlations in the low mercury group also showed significant positive associations with the perceptual performance, fine motor, and working memory scales, whereas the moderate mercury group showed similar results for the numerical and working memory scales.

**Table 4:** Association between four selected McCarthy scales at 5 years of age and 9 oxylipins detected in cord blood plasma for all children (*N* = 25)

	MSCA^a^ scores	Perceptual-performance					Numerical					Fine motor					Working memory				
		Multivariate regression		linear	Spearman correlation		Multivariate regression		linear	Spearman correlation		Multivariate regression		linear	Spearman correlation		Multivariate regression		linear	Spearman correlation	
		Beta	95% CI	*p*	Rho	*p*	Beta	95% CI	*p*	Rho	*p*	Beta	95% CI	*p*	Rho	*p*	Beta	95% CI	*p*	Rho	*p*
Oxylipins (nM)	5,6-DiHET	-8.102	-27.4; 11.2	0.390	-0.115	0.585	5.201	-11.1; 21.5	0.513	0.282	0.171	**-17.56**	-36.2; 1.31	0.066	**-0.381 **	0.060	-2.424	-16.1; 11.8	0.725	0.065	0.759
	8,9-DiHET	-2.625	-11.5; 6.28	0.546	-0.066	0.753	**6.575**	-0.32; 13.5	0.060	**0.386**	0.057	-6.216	-15.2; 2.75	0.164	-0.257	0.214	2.823	-3.62; 9.27	0.371	0.238	0.253
	11,12-DiHET	-1.283	-8.45; 5.88	0.713	-0.031	0.884	**5.324**	-0.18: 10.8	0.057	**0.352**	0.085	-2.615	-10.1; 4.83	0.472	-0.093	0.659	**4.124**	-0.62; 8.87	0.085	**0.408** ^*^	0.043
	14,15-DiHET	-1.420	-8.52; 5.68	0.681	-0.132	0.531	3.040	-2.77; 8.85	0.288	0.162	0.438	-1.998	-9.42; 5.42	0.580	-0.110	0.602	3.779	-0.91; 8.46	0.108	**0.380**	0.061
	TXB_2_	0.933*	0.05; 1.82	0.040	0.395*	0.050	0.301	-0.51; 1.12	0.450	**0.345**	0.091	0.764	-0.21; 1.73	0.116	0.171	0.413	-0.172	-0.88; 0.53	0.615	0.186	0.373
	9,10-DiHOME	0.477	-1.92; 2.87	0.682	0.062	0.767	0.134	-1.88; 2.15	0.891	-0.187	0.371	0.193	-2.33; 2.71	0.874	0.085	0.688	-0.372	-2.10; 1.36	0.657	-0.218	0.294
	12,13-DiHOME	1.003	-2.39; 4.39	0.544	0.200	0.338	-0.015	-2.89; 2.85	0.991	-0.170	0.417	0.945	-2.61; 4.50	0.586	0.232	0.265	-0.744	-3.22; 1.73	0.536	-0.185	0.375
	19,20-DiHDPA	-1.932	-6.98, 3.11	0.434	-0.234	0.261	1.738	-2.49; 5.96	0.401	0.074	0.726	-1.667	-6.99; 3.65	0.521	-0.227	0.275	1.529	-2.06; 5.11	0.383	-0.020	0.924
	14-HDoHE	0.547	-1.20; 2.29	0.520	-0.205	0.327	0.148	-1.33; 1.62	0.836	-0.135	0.519	0.235	-1.61; 2.08	0.793	− 0.245	0.238	-0.296	-1.55; 0.95	0.626	-0.257	0.215

The two statistical models were independently calculated with the same data. Multivariate linear regression model adjusted for cord blood T-Hg concentration, maternal seafood consumption and newborn sex. The four MSCA scales displayed showed significant associations in both models. Association analyses of other MSCA scales are shown in Supplementary Tables 9 A, B

^a^*MSCA* McCarthy Scales of Children’s Abilities. Highlighted significant values (* *p* < 0.05) and borderline values (*p* < 0.1)

### Potential effects of prenatal co-exposure to cadmium and lead

The contribution of co-exposure to cadmium and lead to the changes observed in oxylipin dynamics was examined in pooled samples of cord blood plasma. The analysis of exposure patterns for these metals differed from that of mercury, as indicated by their similar levels in both mercury exposure groups (Table 1). Furthermore, there was a positive correlation between cadmium and lead exposure levels (Spearman rho = 0.355, *p* = 0.040, *N* = 34), but not with T-Hg levels (rho = 0.025, *p* = 0.886 for cadmium, and rho = 0.018, *p* = 0.918 for lead; *N* = 34). This is in agreement with the anticipated main sources of fetal exposure: maternal seafood intake for MeHg, and maternal smoking, for cadmium and lead.

Cadmium and lead did not alter the oxylipin pattern, as indicated by the lack of bivariate correlations between the exposure level of these metals and the concentrations of each of the nine oxylipins detected in cord blood plasma. The Spearman correlations for both metals are shown in Supplementary Table 10, alongside with that of mercury for comparison. Moreover, we used multivariate linear regression models to investigate a contribution of cadmium and lead exposures to the associations between oxylipins and neurodevelopmental scores in the presence of mercury. They were introduced as additional covariates to the previous models. Only minor changes in the significance of some borderline associations were observed (Supplementary Table 11 A, B). Therefore, we could not detect a significant contribution of cadmium and lead co-exposure with MeHg to PUFA signaling.

### Inhibition of soluble epoxide hydrolase induced changes in the oxylipin profile of mouse brain

**Fig. 5:**
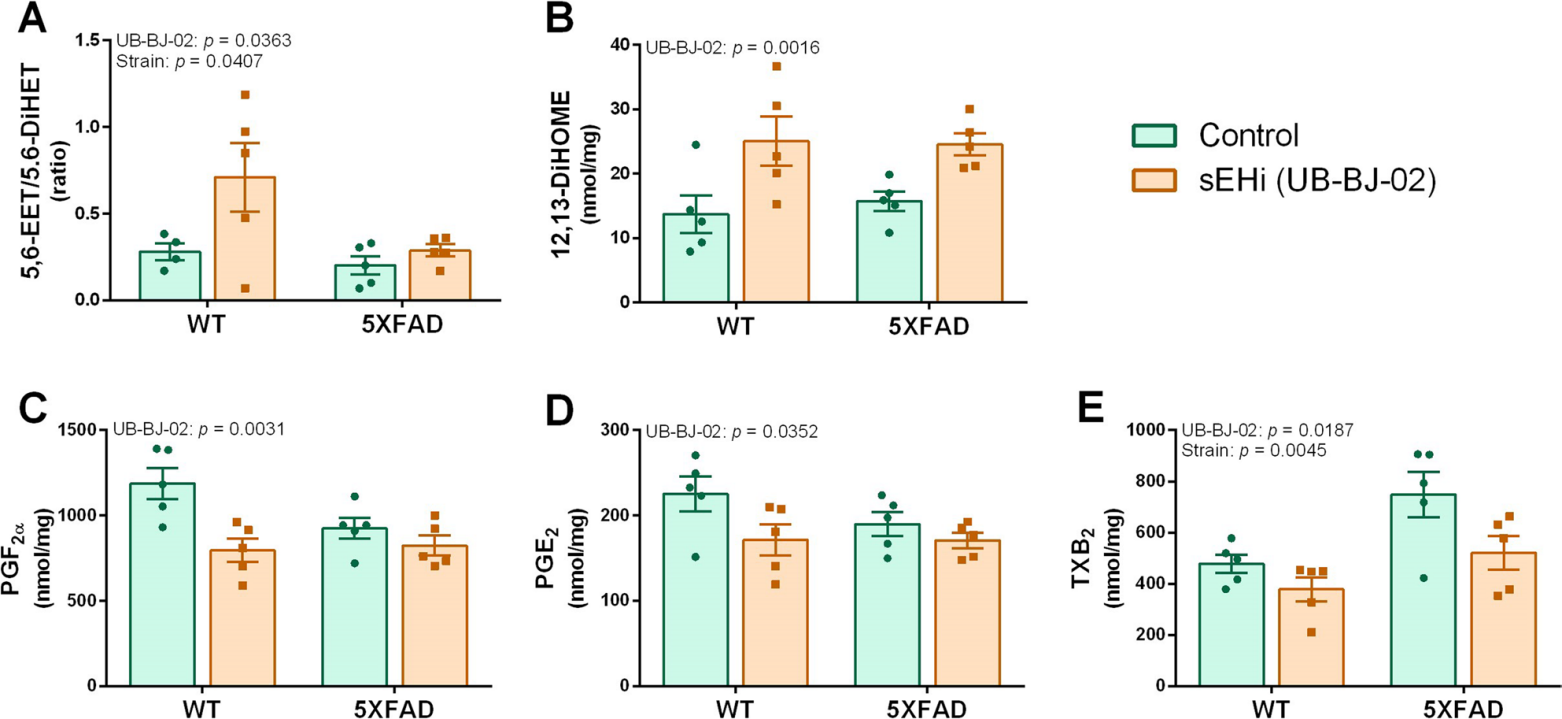
Changes in oxylipin levels in the cerebral cortex of adult mice treated with the sEHi compound UB-BJ-02 for 4 weeks. **A** 5,6-EET/5,6-DiHET ratio. **B** 12,13-DiHOME levels. **C** PGF₂α levels. **D** PGE₂ levels. **E** TXB₂ levels. *N* = 4–5 per group. Statistics: two-way ANOVA, significance of the factors UB-BJ-02 treatment and mouse strain is shown in the graphs

UPLC-MS/MS analysis of the cerebral cortical tissues of WT mice and 5XFAD revealed detectable levels of 13 oxylipins (Supplementary Fig. 2). Both mouse strain and treatment with the sEHi UB-BJ-02 induced changes in the levels of certain oxylipins, as shown in Fig. 5.

The change in the EET/DiHET ratio has been widely used as a measure of the effect of sEH inhibition in various disease models and even in clinical trials [52, 53]. For this reason, the response to sEHi treatment was analyzed as a change in the 5,6-EET/5,6 DiHET ratio. The other three EET isomers had values below LOD level, and their ratios with the corresponding DiHET could not be assessed. Two-way ANOVA showed a significant effect of factor treatment and strain on the 5,6-EET/5,6 DiHET ratio. UB-BJ-02 induced an increase in this ratio, although values were generally lower in the 5XFAD strain (Fig. 5A). Furthermore, a significant increase in 12,13-DiHOME induced by UB-BJ-02 was clearly observed in both WT and 5XFAD mice (Fig. 5B). This oxylipin belongs to the CYP epoxygenase pathway of LA. There was also a significant effect of sEHi in decreasing the pro-inflammatory oxylipins PGF_2α_, PGE_2_, and TXB_2_ from the AA prostaglandin pro-inflammatory pathway (Fig. 5C-E, respectively). Additionally, the 5XFAD strain showed higher levels of TXB_2_ (Fig. 5E). No significant interactions were detected between sEHi treatment and mouse strain.

### Preliminary results on sex-related effects

The potential effects of sex on outcomes associated with low and moderate prenatal mercury exposure could not be reliably assessed due to the small number of cases of each sex in most groups. Nevertheless, an exploratory two-way ANOVA in cord-blood plasma data suggested a sex-related modulation in some oxylipin levels (Supplementary Table 13) and McCarthy scale scores (Supplementary Table 14). These results should be interpreted with caution and investigated in future studies with larger sample sizes. Likewise, sex-related differences in the mice oxylipin profiles were not analyzed.

## Discussion

**Fig. 6:**
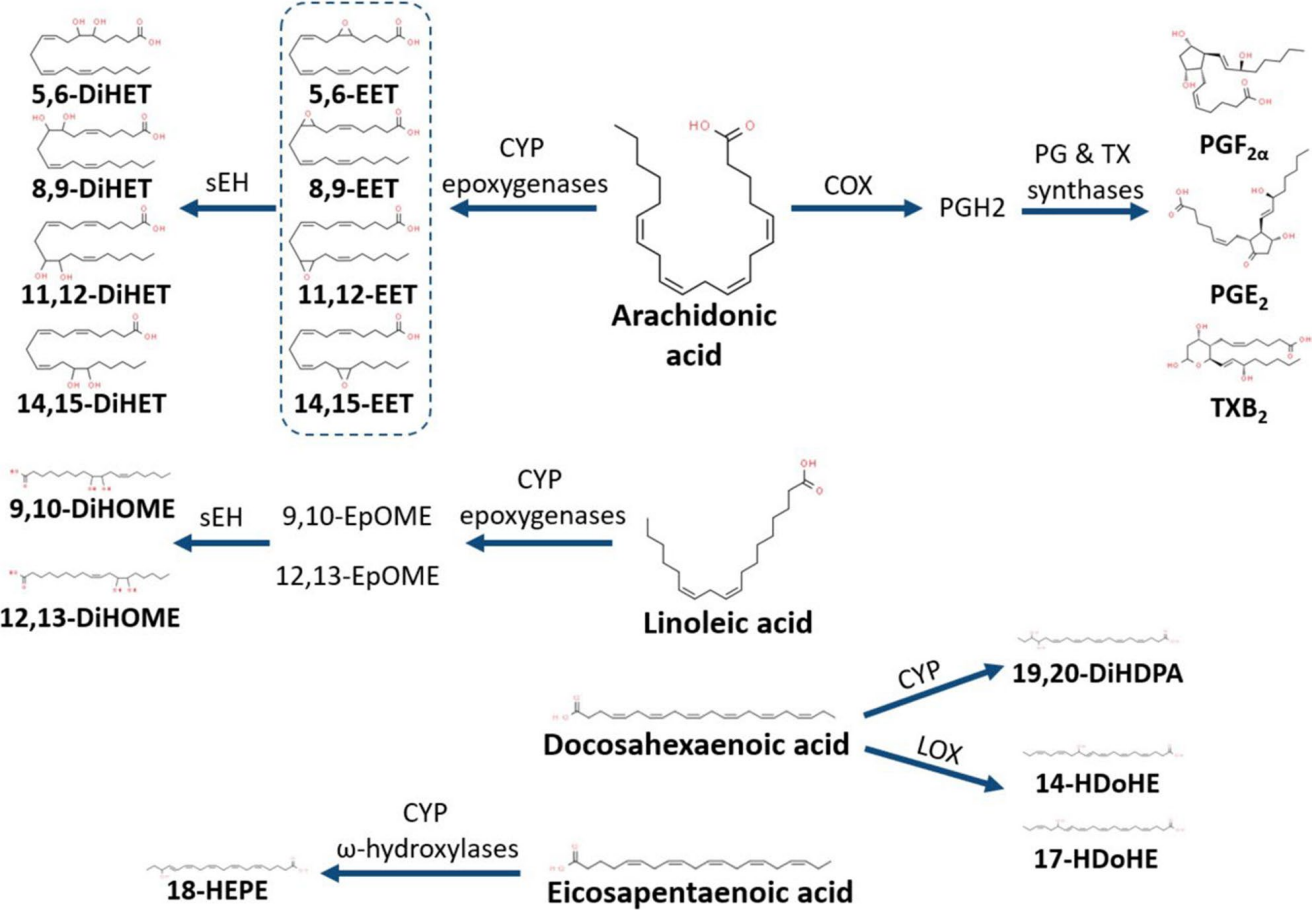
Oxylipins relevant to the study and the corresponding metabolic pathway from their respective parent polyunsaturated fatty acids

The analysis of the oxylipin profile in placental tissue and cord blood plasma, along with their association with children’s neurodevelopment scores, revealed significant changes linked to prenatal mercury exposure in this cohort from Valencia, Spain. Bioactive lipids indicators of metabolic and inflammatory pathways have been proposed as prenatal biomarkers of exposure to environmental toxins [54]. Prenatal PUFA signaling induced by mercury has not been previously analyzed. The use of placenta or cord blood instead of maternal blood is relevant, as mercury shows a higher affinity for fetal proteins [55]. The metabolic pathways of the oxylipins that showed changes in the human and mouse tissues analyzed are displayed in Fig. 6.

Children in the moderate prenatal mercury exposure group of this study had cord blood T-Hg levels that were 3- to 8-fold higher than the equivalent of the proposed US-EPA RfD for MeHg intake [19]. These moderately high levels remained well below those reported in cases of mercury intoxication, and no impairments were detected in the neurodevelopmental scores. However, we cannot discard an increased neurotoxic risk, as suggested by the mild deleterious changes observed in placental biomarkers. The partial inhibition of proteasome activity may have reduced the degradation of abnormal intracellular proteins and subsequent protein turnover. Mercury is a known dose-dependent inhibitor of proteasome activity [56]. Regarding the analysis of epigenetic changes, increased trymethylation of histone H3K27 may have induced specific gene silencing during early development [57]. Placental DNA methylation changes induced by prenatal mercury exposure have been associated with adverse infant neurodevelopment [58]. Furthermore, experimental studies have revealed histone modifications induced by MeHg [59], including increased H3K27me3 [60]. Previous studies involving large cohorts of children from the INMA project across various Spanish regions have identified cognitive and motor score impairments at ages 4–5 years associated with mercury in placental tissue [25], as well as poorer psychomotor development in girls at 14 months of age associated with cord blood mercury levels [47]. However, another analysis of these cohorts has shown that a doubling of cord blood mercury levels was associated with higher scores in most of the neuropsychological scales at 4–5 years of age. Notably, this association was found negative in children whose mothers consumed fewer than three fish servings per week during pregnancy [30]. These findings highlight the relevance of fish consumption in protecting against prenatal mercury exposure in this population. Therefore, a maternal diet rich in fish and other PUFA-containing nutrients may mitigate the neurodevelopmental risks associated with prenatal mercury exposure.

The relative increase found in the levels of some anti-inflammatory and protective oxylipins in the placenta or cord blood plasma of the moderate mercury versus low mercury groups may be related to an increase in PUFA-derived signaling to protect against MeHg damage. These changes were probably reproduced in the fetal central nervous system and other tissues. The increase of DiHETs levels in the placenta may be attributed to a higher generation of their potent neuroprotective precursors, EETs, which undergo rapid metabolization. DiHETs are the stable final metabolites of the anti-inflammatory pathway initiated by CYP epoxygenation of AA. Their anti-inflammatory activity is relatively low, but their increase suggests activation of AA-associated anti-inflammatory activity. In the mouse study, chronic dosing with the sEHi UB-BJ-02 induced an increase in the EET/DiHET ratio, as expected. The treatment decreased brain levels of the most abundant pro-inflammatory oxylipins (PGF_2α_, PGE_2_, and TXB_2_), supporting the benefits of sEHi treatment previously reported in developing TgAD mice [38] and in adult TgAD mice [61, 62]. However, the EET/DiHET ratio increase was less pronounced in the 5XFAD strain than in the WT strain. We can speculate that there is an increased use and degradation of EETs in the TgAD mouse brain. In the cord blood plasma of children, an increase of 19,20-DiHDPA, a stable final metabolite of DHA, was found in the moderate mercury exposure group, indicating mobilization of DHA, which also protects against toxic damage. Accordingly, the increase of 19,20-DiHDPA and other DHA metabolites in mouse pup brain has been related to the neuroprotection afforded by maternal DHA supplementation against MeHg [63]. Interestingly, the sEHi treatment in the mouse study also modulated the metabolism of LA epoxy metabolites, leading to increased 12,13-DiHOME. This oxylipin is a bioactive compound involved in metabolic health effects [64].

Children’s neurodevelopment scores at 14 months of age were positively associated with levels of AA-derived EET and DiHET isomers across tissue samples and statistical analyses. In placenta samples, positive Spearman correlation between several of these oxylipins and the Bayley mental scale scores in the low mercury exposure group were maintained in the multivariate linear regression analysis of pooled samples adjusted for mercury levels and other variables. In cord-blood plasma samples, positive correlations between DiHETs and Bayley mental scale scores in the low mercury exposure group were also maintained in the adjusted linear regression model. This confirms the beneficial impact of the AA epoxide metabolites in the neurodevelopment of cognitive substrates. MeHg is primarily neurotoxic for the granule cell neurons of the cerebellum, which affects motor areas during brain development and contributes to cognitive impairment [6, 65, 66]. Therefore, fine motor activities in children may be highly sensitive to the crosstalk signaling between mercury exposure and PUFA status [30]. However, the analysis of associations between oxylipins and psychomotor neurodevelopment yielded less consistent results across tissue samples. Regarding DHA-derived oxylipins, levels of its final metabolite 19,20-DiHDPA showed a positive association with Bayley mental scale scores in the pooled sample analysis, further supporting its beneficial neurodevelopmental effects.

At the older age of 5 years, only cord blood donors could be evaluated for McCarthy scores, as previously indicated. At this age, associations between anti-inflammatory oxylipins and neurodevelopmental scores were less evident than at 14 months. However, levels of some AA-derived DiHETs were positively associated by Spearman correlations with the McCarthy working memory scale in the moderate mercury group. The association showed a trend after adjustment for confounders in the regression model of pooled samples. These differential associations according to the children’s age suggest that prenatal PUFA activation would progressively counteract neurodevelopment delays induced by prenatal MeHg exposure. Therefore, we can speculate that the positive associations between EETs and DiEHTs of AA, or other beneficial oxylipins, with neurodevelopment scores up to 5 years of age reflect long-lasting effects of fetal PUFA signaling.

Finally, some positive associations found between neurodevelopmental scores and harmful pro-inflammatory oxylipins may be circumstantial. This applies to TXB_2_, a final metabolite of thromboxane A_2_, whose synthesis has been shown to be induced by MeHg in vivo and in vitro in endothelial cells and activated platelets [67, 68]. TXB_2_ in cord blood plasma showed positive Spearman correlations with some neurodevelopmental scores in the moderate mercury exposure group at both 14 months and 5 years of age. Positive association of TXB_2_ with McCarthy scales were confirmed for the perceptual-performance scale after adjustment for confounders in the analysis of pooled cord blood plasma data. Therefore, a potential pro-inflammatory effect of MeHg through thromboxane A_2_ - TXB_2_ may coexist with the neurodevelopmental benefits conferred by nutrients associated with higher fish consumption. Moreover, elevated maternal fish intake may itself favor thromboxane synthesis through increased availability of its substrate AA [69]. Basal platelet activation with increased thromboxane biosynthesis during pregnancy [70] may also contribute to this response. Other compensatory vascular adaptation cannot be ruled out. In addition, co-exposure of MeHg with cadmium and lead, two highly neurotoxic pollutants [43, 71–73], did not induce further changes in the associations between neurodevelopmental scores and PUFA metabolic pathways.

**Fig. 7:**
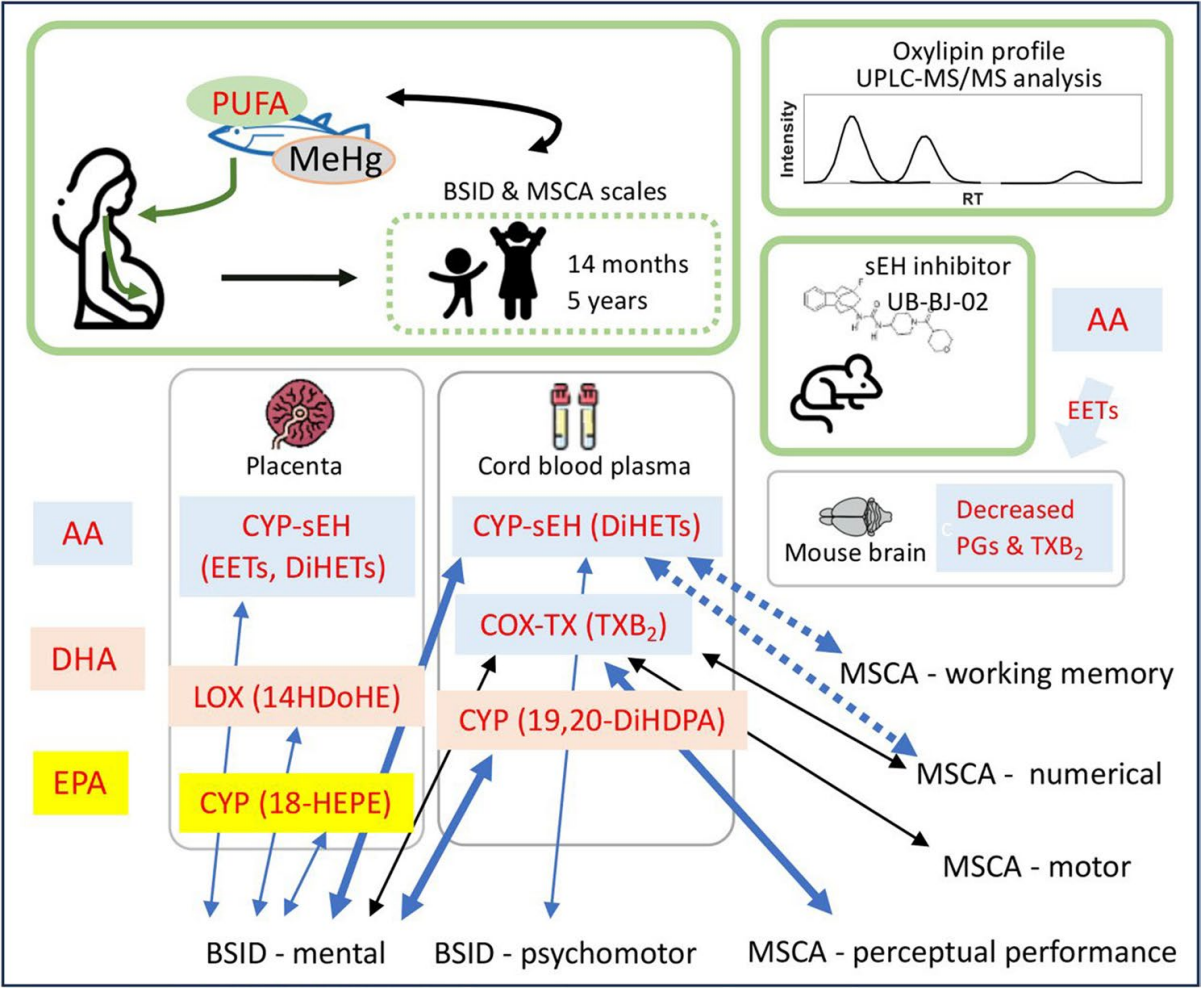
Schematic representation of the main findings on prenatal MeHg exposure and polyunsaturated fatty acid (PUFA) signaling during children neurodevelopment. The upper panels show the study design. Activated PUFA metabolic pathways and their detected oxylipins are indicated for arachidonic acid (AA), docosahexaenoic acid (DHA), and eicosapentaenoic acid (EPA) in placenta and cord blood plasma. Double-headed arrows indicate positive associations between oxylipins and specific neurodevelopmental scores assessed by the Bayley Scales of Infant and Toddler Development (BSID) and the McCarthy Scales of Children’s Abilities (MSCA). The upper right shows the mechanistic study in mice dosed with a soluble epoxide hydrolase (sEH) inhibitor. Symbols: thin arrows indicate Spearman correlations under low (blue) or moderate (black) mercury exposure; thick blue arrows: multivariate linear regression using pooled samples; dashed arrows: borderline statistical significance

This study focused on PUFA dynamics in a fish-eating population, and other protective nutrients such as selenium were not assessed. Further studies with well characterized dietary patterns with respect to mercury content would help to disentangle the effects of MeHg from those derived from higher nutrient intake among frequent fish consumers [74]. A summary of the study design and main results is shown in Fig. 7.

Limitations and strengths: The first limitation is the relatively small sample size, particularly for placenta data, which reduced the statistical power of the analysis. Therefore, some tests and association models were only applied to pooled plasma samples. Nevertheless, since placenta samples were collected homogeneously in paired fetal - maternal tissues, their findings are sufficiently robust for exploratory purposes. Another limitation is the use of different donors for placenta and cord blood samples, which prevented direct comparison between sample types and limited conclusions about prenatal mercury exposure. As is known, the placenta acts as a reservoir for both beneficial nutrients and toxic chemicals, reflecting chronic exposure during pregnancy, whereas cord blood indicates current levels of toxins and metabolites. The main strength of the study is the availability of tissue samples collected from a birth cohort with prospectively performed neurodevelopmental assessments. In addition, the use of brain samples from mice treated with a pathway-modifying agent enables further discussion on specific oxylipins. Finally, this multi-approach design showed consistent patterns across independent datasets, reinforcing the robustness of the findings despite the inherent limitation of small sample size in some study groups.

## Conclusions

Overall, our findings on prenatal PUFA-derived signaling in children exposed to two levels of mercury support the neurodevelopmental benefits of a maternal diet rich in EPA, DHA, and AA. Despite the absence of postnatal deficits in neurodevelopmental scores at 14 months and 5 years in the higher mercury exposure group of this cohort, a substantial number of statistical associations between children’s scores and oxylipin levels in placental tissue or cord blood plasma suggest a protective role of PUFAs. Oxylipin dynamics highlight the potentially beneficial role of AA-derived epoxides, supported by their anti-inflammatory effect observed in the adult mouse brain. Therefore, AA may be considered alongside the better-known DHA and EPA as a first-line PUFA to promote fetal brain development and resilience against neurotoxic damage. Further studies with larger cohorts are warranted to confirm these results and to evaluate the effectiveness of maternal nutrition interventions in mitigating the risks associated with prenatal exposure to MeHg and other environmental neurotoxicants.

## Supplementary Information

Supplementary Material 1: Experimental procedures for oxylipin sample preparation and analysis. Supplementary tables and figures (Supplementary Table 1: Placenta and cord blood samples used in the study. Supplementary Table 2: Acquisition parameters of the UPLC-MS/MS method. Supplementary Table 3: Oxylipins detected in the tissue samples of the study. Supplementary Fig. 1: Proteasome activities and histone modifications of placental tissue. Supplementary Table 4: Scores of McCarthy scales of neurodevelopment at 5 years of age in donors of placental samples. Supplementary Table 5: Spearman correlations between Bayley scales and oxylipins in placental tissue. Supplementary Table 6: Spearman correlations between Bayley scales and oxylipins in cord blood plasma. Supplementary Table 7: Spearman correlations between McCarthy scales and oxylipins in cord blood plasma. Supplementary Table 8: Spearman correlations between McCarthy scales and oxylipins in placental tissue for all children. Supplementary Table 9 A, B: Multivariate linear regression and Spearman correlations between McCarthy scales and oxylipins in cord blood plasma for all children (selected scales in Table 4 of main text). Supplementary Fig. 2: Oxylipin profile in cerebral cortical tissue of WT and 5XFAD mouse group after sEHi treatment. Supplementary Table 10: Spearman correlations between cadmium and lead and oxylipins in cord blood samples. Table Supplementary 11 A, B: Multivariate linear regression between neurodevelopmental BSID and MSCA scales adjusted for cadmium and lead exposure. Supplementary Table 12: Mean oxylipins levels in cord blood plasma of female and male groups. Supplementary Table 13: Mean McCarthy scores in female and male groups of cord blood plasma donors).

## Data Availability

Raw data not included in the main text or supplementary materials will be made available on request.

## Abbreviations

AA: Arachidonic acid
BMI: Body mass index
BSID: Bayley Scales of Infant and Toddler Development III
COX: Cyclooxygenase
CYP: Cytochrome P450
DHA: Docosahexaenoic acid
19,20-DiHDPA: 19,20-Dihydroxydocosapentaenoic acid
9,10-DiHOME: 9,10-Dihydroxy-12Z-octadecenoic acid
12,13-DiHOME: 12,13-Dihydroxy-9Z-octadecenoic acid
DiHET: Dihydroxieicosatrienoic acid (5,6-, 8,9-, 11,12-, 14,15-DiHET)
EPA: Eicosapentaenoic acid
EET: Epoxyeicosatrienoic acid (5,6-, 8,9-, 11,12-, 14,15-EET)
18-HEPE: 18-Hydroxyeicosapentaenoic acid
LA: Linoleic acid
LOX: Lipoxygenase
MeHg: Methylmercury
MSCA: McCarthy Scales of Children’s Abilities
PGF_2α_: Prostaglandin F2 alpha
PGE_2_: Prostaglandin E2
PUFA: Polyunsaturated fatty acid
sEH: Soluble epoxide hydrolase enzyme
sEHi: sEH inhibitor
T-Hg: Total mercury
TgAD: Transgenic Alzheimer’s disease mouse
TXB_2_: Thromboxane B2
UPLC-MS/MS: Ultraperformance liquid chromatography coupled to tandem mass spectrometry
US-EPA RfD: Reference Dose established by the U.S. Environmental Protection Agency
WT: Wild-type mouse
5XFAD: TgAD, strain 5XFAD
