# Supplementary material for: Activation of anti-inflammatory pathways by polyunsaturated fatty acid signaling may protect neurodevelopment in children prenatally exposed to methylmercury
Source: Environ Health. 2026 Apr 16;25:36. doi: 10.1186/s12940-026-01270-2 (PMC13085584; doi:10.1186/s12940-026-01270-2)
Supplement: Supplementary file 1 — Supplementary Material 1: Experimental procedures for oxylipin sample preparation and analysis. Supplementary tables and figures (Supplementary Table 1: Placenta and cord blood samples used in the study. Supplementary Table 2: Acquisition parameters of the UPLC-MS/MS method. Supplementary Table 3: Oxylipins detected in the tissue samples of the study. Supplementary Fig. 1: Proteasome activities and histone modifications of placental tissue. Supplementary Table 4: Scores of McCarthy scales of neurodevelopment at 5 years of age in donors of placental samples. Supplementary Table 5: Spearman correlations between Bayley scales and oxylipins in placental tissue. Supplementary Table 6: Spearman correlations between Bayley scales and oxylipins in cord blood plasma. Supplementary Table 7: Spearman correlations between McCarthy scales and oxylipins in cord blood plasma. Supplementary Table 8: Spearman correlations between McCarthy scales and oxylipins in placental tissue for all children. Supplementary Table 9 A, B: Multivariate linear regression and Spearman correlations between McCarthy scales and oxylipins in cord blood plasma for all children (selected scales in Table 4 of main text). Supplementary Fig. 2: Oxylipin profile in cerebral cortical tissue of WT and 5XFAD mouse group after sEHi treatment. Supplementary Table 10: Spearman correlations between cadmium and lead and oxylipins in cord blood samples. Table Supplementary 11 A, B: Multivariate linear regression between neurodevelopmental BSID and MSCA scales adjusted for cadmium and lead exposure. Supplementary Table 12: Mean oxylipins levels in cord blood plasma of female and male groups. Supplementary Table 13: Mean McCarthy scores in female and male groups of cord blood plasma donors). [file 12940_2026_1270_MOESM1_ESM.docx]

**SUPPLEMENTARY INFORMATION**

Activation of anti-inflammatory pathways by polyunsaturated fatty acid signaling may protect neurodevelopment in children prenatally exposed to methylmercury

Clara Bartra, Sabrina Llop, Julia Kuligowski, Abel Albiach-Delgado, Cristina Suñol, Eduard Rodríguez-Farré, Ferran Ballester, Raquel Soler-Blasco, Beatrice Jora, Júlia Jarné-Ferrer, Christian Griñán-Ferré, Santiago Vázquez, Mercè Pallàs, Coral Sanfeliu

**Number of pages: 22**

**Number of figures: 2**

**Number of tables: 13**

**EXPERIMENTAL PROCEDURES FOR OXYLIPIN SAMPLE PREPARATION AND ANALYSIS**

**Materials, Standards, and Reagents**

LC-MS grade solvents, including methanol (CH₃OH) and acetonitrile (CH₃CN), were purchased from J.T. Baker (Phillipsburg, NJ, USA). Formic acid (HCOOH, 98%) was obtained from Panreac (Barcelona, Spain). Ultrapure water (H₂O) was produced using a Milli-Q system from Merck Millipore (Darmstadt, Germany).

Standards for oxylipins were sourced from Cayman Chemical (Michigan, USA). 3,5-Di-tert-4-butylhydroxytoluene (BHT) was acquired from Merck Millipore (Darmstadt, Germany), and ammonium formate was supplied by VWR Chemicals (Radnor, Pennsylvania, USA). External calibration lines were prepared via serial dilution from appropriate stock and working solutions in a 50:50 (v/v) mixture of methanol and acetonitrile covering a range between 0.4 and 225 nM.

**Sample Preparation and Analysis**

All samples were stored at -80°C until analysis. For tissue homogenization, cortex and placenta samples were thawed on ice, and approximately 20 mg of each sample was mixed with 0.1 % ammonium acetate (20 μL/mg of tissue). The samples were homogenized using a Precellys Evolution® mixer (3 cycles of 20 seconds at 6500 rpm at 5°C) and centrifuged for 11 min at 13000 rpm and 4°C.

For sample treatment, 200 μL of the supernatant from the tissues and plasma samples were spiked with 5 μL of 10 mg/mL BHT in ethanol and 5 μL of an internal standard solution containing various oxylipins: 0.5 μM of (±)12(13)-DiHOME-d_4_, (±)9(10)-DiHOME-d_4_, (±)14(15)-DiHET-d_11_, 12(S)-HETE-d_8_, and Leukotriene B_4_-d_4_ and 1.25 μM of PGF_2α_-d_4_, Maresin 2-d_5_, Lipoxin A_4_ - d_5_, Resolvin D_2_ -d_5_, (±)8(9)-DiHET-d_11_, (±)11(12)-EET-d_11_, (±)8(9)-EET-d_11_, (±)11(12)-DiHET-d_11_, (±)14,15-EET-d_11_, and (±)5,6-EET-d_11_. Samples were vortexed for 15 min at 4°C and centrifuged for 15 min at 3500 g at 4°C. The supernatant was then diluted to 1400 μL with H₂O.

Oxylipins were extracted and pre-concentrated using solid-phase extraction (SPE) with Oasis-HLB 96-well plates (30 mg) from Waters (Milford, MA, USA). To perform solid-phase extraction (SPE), the PlatePrep 96-well Vacuum Manifold from Merck KGaA (Darmstadt, Germany) was used. The plate wells were conditioned sequentially with 1 mL of ethyl acetate, 1 mL of methanol, and 1 mL of H₂O (containing 0.1 % acetic acid and 5% methanol. Samples were loaded and washed with 1 mL of H₂O (containing 0.1% acetic acid and 5% methanol) and eluted using 250 μL of methanol followed by three washes with 250 μL of ethyl acetate each. The recovered extracts were evaporated using a miVac Concentrator from SP Scientific (Gardiner, NY, USA), then reconstituted in 50 μL of a 50:50 (v/v) mixture of methanol and acetonitrile.

Sample extracts were analyzed with an Acquity-Xevo TQ-XS system from Waters (Milford, MA, USA) operating in the negative electrospray ionization mode (ESI-). Separations were carried out on a Waters Acquity UPLC BEH C18 column (2.1 x 100 mm, 1.7 μm) using a binary gradient consisting of 0.1% v/v acetic acid (channel A) and a 90:10 (v/v) mixture of acetonitrile and isopropanol (channel B). Flow rate, column temperature, and injection volume were set at 0.6 mL min^-1^, 40 °C, and 5 µL, respectively. The gradient with a total run time of 10.0 min was as follows: from 0.0 to 1.0 min, 75% v/v of channel B; from 1.0 to 8.0 min %B increased up to 95%; from 8.0 to 8.5 min %B conditions were held constant at 95% B followed by the return to initial conditions (i.e., 75% B) between 8.51 and 10.0 min; conditions were maintained for 1.5 min for system re-equilibration. The source and desolvation temperatures were set to 150°C and 600°C, respectively, with nitrogen cone and desolvation gas flows at 150 L/h and 1000 L/h, respectively. The dwell time was adjusted to ensure a minimum of 10 data points per peak. Multiple reaction monitoring (MRM) conditions are summarized in Supplementary Table 2.

**Data Analysis**

Raw chromatograms were analyzed using MassLynx v4.2 software from Waters (Milford, MA, USA), which included peak area integration and interpolation in external calibration lines.

**Oxylipins quantified in the study**

The levels of some oxylipins were below the limit of detection and could not be quantified in one or more of the tissue types. Of the 32 oxylipins analyzed, a total of 17 oxylipins was quantified in placental tissues, 9 in cord blood plasma, and 13 in mouse cortical brain tissue. The oxylipins used in the study are described in Supplementary Table 3.

**SUPPLEMENTARY TABLES AND FIGURES**

**Supplementary Table 1** Placenta and cord blood samples used in the study.

| Sample ID | Sex | Sample Type | T-Hg (µg/L)^1^ | Exposure group^2^ |
| --- | --- | --- | --- | --- |
| 2 | Male | Pairs of fetal and maternal placenta | 4.8 | Low |
| 399 | Female | Pairs of fetal and maternal placenta | 5.7 | Low |
| 457 | Male | Pairs of fetal and maternal placenta | 6.4 | Low |
| 477 | Male | Pairs of fetal and maternal placenta | 3.8 | Low |
| 908 | Female | Pairs of fetal and maternal placenta | 2.9 | Low |
| 933 | Male | Pairs of fetal and maternal placenta | 2.3 | Low |
| 166 | Female | Pairs of fetal and maternal placenta | 20 | Moderate |
| 489 | Male | Pairs of fetal and maternal placenta | 29 | Moderate |
| 822 | Female | Pairs of fetal and maternal placenta | 24 | Moderate |
| 866 | Male | Pairs of fetal and maternal placenta | 23 | Moderate |
| 887 | Female | Pairs of fetal and maternal placenta | 27 | Moderate |
| 893 | Male | Pairs of fetal and maternal placenta | 20 | Moderate |
| 61 | Female | Cord blood plasma | 6.6 | Low |
| 84 | Male | Cord blood plasma | 3.4 | Low |
| 103 | Male | Cord blood plasma | 6.4 | Low |
| 199 | Female | Cord blood plasma | 5.1 | Low |
| 232 | Male | Cord blood plasma | 5.5 | Low |
| 351 | Male | Cord blood plasma | 1.4 | Low |
| 358 | Female | Cord blood plasma | 1.4 | Low |
| 434 | Female | Cord blood plasma | 1.4 | Low |
| 470 | Female | Cord blood plasma | 1.4 | Low |
| 493 | Female | Cord blood plasma | 1.4 | Low |
| 513 | Male | Cord blood plasma | 1.4 | Low |
| 527 | Female | Cord blood plasma | 1.4 | Low |
| 532 | Male | Cord blood plasma | 1.4 | Low |
| 604 | Female | Cord blood plasma | 1.4 | Low |
| 614 | Female | Cord blood plasma | 1.4 | Low |
| 617 | Female | Cord blood plasma | 1.4 | Low |
| 807 | Male | Cord blood plasma | 1.4 | Low |
| 840 | Male | Cord blood plasma | 1.4 | Low |
| 844 | Male | Cord blood plasma | 1.4 | Low |
| 936 | Male | Cord blood plasma | 2 | Low |
| 116 | Male | Cord blood plasma | 21 | Moderate |
| 142 | Male | Cord blood plasma | 40 | Moderate |
| 203 | Female | Cord blood plasma | 32 | Moderate |
| 214 | Male | Cord blood plasma | 33 | Moderate |
| 240 | Male | Cord blood plasma | 23 | Moderate |
| 311 | Male | Cord blood plasma | 64 | Moderate |
| 418 | Male | Cord blood plasma | 57 | Moderate |
| 485 | Male | Cord blood plasma | 44 | Moderate |
| 541 | Male | Cord blood plasma | 66 | Moderate |
| 608 | Female | Cord blood plasma | 42 | Moderate |
| 616 | Male | Cord blood plasma | 64 | Moderate |
| 645 | Female | Cord blood plasma | 40 | Moderate |
| 674 | Male | Cord blood plasma | 40 | Moderate |
| 760 | Male | Cord blood plasma | 51 | Moderate |
| 779 | Male | Cord blood plasma | 43 | Moderate |
| 865 | Male | Cord blood plasma | 41 | Moderate |
| 886 | Female | Cord blood plasma | 41 | Moderate |
| 899 | Male | Cord blood plasma | 43 | Moderate |
| 928 | Female | Cord blood plasma | 45 | Moderate |

Notes: ^1^T-Hg, total mercury measured in whole cord blood of the donors. ^2^Low and moderate exposure groups include samples with T-Hg 1.4 - 6.6 µg/L and 20 - 66 µg/L, respectively.

**Supplementary Table 2** Acquisition parameters of the UPLC-MS/MS method.

| **Analyte** | **Precursor PUFA** | ***m/z*** | **Cone (V)** | **CE (eV)** | ***m/z*** | **RT (min)** | **Internal**  **Standard** |
| --- | --- | --- | --- | --- | --- | --- | --- |
|  |  | **Precursor Ion** |  |  | **Daughter Ion** |  |  |
| **PGF_2α_** | AA | 353.28 | 40 | 25 | 193.08 | 3.19 | PGF_2α_-d_4_ |
| **PGE_2_** | AA | 351.24 | 20 | 15 | 271.15 | 3.26 | PGF_2α_-d_4_ |
| **17,18-DiHETE** | EPA | 335.24 | 30 | 20 | 247.09 | 4.40 | 14,15-DiHETrE-d_11_ |
| **Resolvin D_5_** | DHA | 359.27 | 30 | 15 | 119.11 | 4.43 | Maresin 2 -d_5_ |
| **14,15-DiHETE** | EPA | 335.26 | 20 | 20 | 207.05 | 4.52 | 14,15-DiHETrE-d_11_ |
| **8(S),15(S)-DiHETE** | AA | 335.25 | 40 | 20 | 127.01 | 4.52 | 14,15-DiHETrE-d_11_ |
| **12,13-DiHOME** | LA | 313.25 | 40 | 20 | 183.07 | 4.64 | 12,13-DiHOME-d_4_ |
| **Maresin 2** | DHA | 359.2 | 20 | 20 | 177.15 | 4.75 | Maresin 2 -d_5_ |
| **9,10-DiHOME** | LA | 313.26 | 20 | 20 | 201.03 | 4.78 | 9,10-DiHOME-d_4_ |
| **19,20-DiHDPA** | DHA | 361.31 | 30 | 15 | 273.15 | 4.85 | 14,15-DiHETrE-d_11_ |
| **17-HDHA** | DHA | 343.2 | 35 | 15 | 281.3 | 5.69 | 12-HETE-d_8_ |
| **14-HDHA** | DHA | 343.2 | 35 | 15 | 205 | 5.79 | 12-HETE-d_8_ |
| **TXB_2_** | AA | 369.27 | 20 | 15 | 169.04 | 2.89 | 14,15-DiHETrE-d_11_ |
| **LTB_4_** | AA | 335.23 | 20 | 15 | 195 | 4.52 | LTB_4_-d_4_ |
| **PDX** | DHA | 359.50 | 20 | 20 | 153.04 | 4.39 | 12-HETE-d_8_ |
| **Maresin 1** | DHA | 359.26 | 10 | 15 | 177.15 | 4.44 | Maresin 2 -d_5_ |
| **Neuroprotectin D_1_ \| PD-1** | DHA | 359.25 | 35 | 15 | 153.09 | 4.33 | 12-HETE-d_8_ |
| **18-HEPE** | EPA | 317.23 | 30 | 10 | 299.09 | 5.16 | 12-HETE-d_8_ |
| **LXA4 \| 5(S),6(R),15(S)-TriHETE** | AA | 351.20 | 35 | 15 | 115.10 | 3.64 | Lipoxin A_4_-d_5_ |
| **Lipoxin B_4_** | AA | 351.20 | 35 | 15 | 221.20 | 3.33 | Lipoxin A_4_-d_5_ |
| **Resolvin D_3_** | DHA | 375.10 | 30 | 15 | 147.00 | 3.30 | Resolvin D_2_-d_5_ |
| **Resolvin D_1_** | DHA | 375.28 | 10 | 15 | 141.03 | 3.60 | 14,15-DiHETrE-d_11_ |
| **Resolvin E_2_** | EPA | 375.28 | 35 | 20 | 141.03 | 3.60 | Resolvin D_2_-d_5_ |
| **Resolvin E_1_** | EPA | 349.20 | 35 | 15 | 195.00 | 2.36 | 14,15-DiHETrE-d_11_ |
| **5,6-DiHETrE** | AA | 337.2 | 35 | 15 | 145.1 | 5.39 | 8,9-DiHETrE-d_11_ |
| **8,9-DiHETrE** | AA | 337.2 | 35 | 15 | 127 | 5.16 | 8,9-DiHETrE-d_11_ |
| **11,12-DiHETrE** | AA | 337.2 | 35 | 15 | 167.2 | 4.99 | 11,12-DiHETrE-d_11_ |
| **14,15-DiHETrE** | AA | 337.26 | 20 | 15 | 207.05 | 4.83 | 11,12-DiHETrE-d_11_ |
| **14,15-EET** | AA | 319.2 | 35 | 15 | 219.2 | 6.12 | Resolvin D_2_-d_5_ (cortex) / 14,15-DiHETrE-d_11_ (placenta) / 12-HETE-d_8_ (plasma) |
| **5,6- EET** | AA | 319.2 | 35 | 15 | 191.2 | 6.43 | Resolvin D_2_-d_5_ (cortex, placenta) / 5,6-EET-d_11_ (plasma) |
| **11,12- EET** | AA | 319.2 | 35 | 15 | 167.1 | 6.29 | Resolvin D_2_-d_5_ (cortex) / PGF_2α_-d_4_ (placenta) / 12-HETE-d_8_ (plasma) |
| **8,9- EET** | AA | 319.2 | 35 | 15 | 155.1 | 6.36 | 8,9- EET -d_11_ |
| **Lipoxin A_4_-d_5_** | - | 356.2 | 35 | 20 | 115 | 3.62 | - |
| **Resolvin D_2_-d_5_** | - | 380.5 | 35 | 15 | 141 | 3.36 | - |
| **PGF_2α_-d_4_** | - | 357.3 | 35 | 15 | 197.2 | 3.19 | - |
| **LTB_4_-d_4_** | - | 339.2 | 35 | 15 | 197.1 | 4.50 | - |
| **12,13-DiHOME-d_4_** | - | 317.26 | 20 | 20 | 185.06 | 4.62 | - |
| **Maresin 2-d_5_** | - | 364.5 | 20 | 20 | 177.06 | 4.74 | - |
| **9,10-DiHOME-d_4_** | - | 317.26 | 30 | 25 | 203.4 | 4.75 | - |
| **14,15-DiHETrE-d_11_** | - | 348.34 | 40 | 15 | 207.05 | 4.79 | - |
| **11,12-DiHETrE-d_11_** | - | 348.2 | 25 | 20 | 167.2 | 4.97 | - |
| **8,9-DiHETrE-d_11_** | - | 348.2 | 25 | 22 | 127 | 5.13 | - |
| **12-HETE-d_8_** | - | 343.2 | 20 | 15 | 281.3 | 5.8 | - |
| **5,6- EET -d_11_** | - | 330.2 | 45 | 15 | 202.2 | 6.4 | - |
| **11,12- EET -d_11_** | - | 330.2 | 45 | 16 | 167.1 | 6.25 | - |
| **8,9- EET -d_11_** | - | 330.2 | 42 | 16 | 268.1 | 6.32 | - |

**Supplementary Table 3** Oxylipins detected in the tissue samples of the study.

| **Abbreviation** | **Full name** | **Tissue** | **Characteristics^1^** |
| --- | --- | --- | --- |
| **Arachidonic acid (AA) metabolites obtained through the cytochrome P450 epoxygenase pathway** | | | |
| 5,6- EET | 5,6-Epoxyeicosatrienoic acid | Human placenta  Mouse brain | Potent anti-inflammatory isomers, rapidly degraded to DiHETs by the soluble epoxyde hydrolase enzyme. |
| 8,9- EET | 8,9-Epoxyeicosatrienoic acid | Human placenta |  |
| 11,12- EET | 11,12-Epoxyeicosatrienoic acid | Human placenta |  |
| 14,15-EET | 14,15-Epoxyeicosatrienoic acid | Human placenta |  |
| 5,6-DiHETrE \| 5,6-DiHET | 5,6-Dihydroxyeicosatrienoic acid | Human placenta  Human cord blood plasma  Mouse brain | Stable final metabolites of their respective EET isomer, with low anti-inflammatory activity. |
| 8,9-DiHETrE \| 8,9-DiHET | 8,9-Dihydroxyeicosatrienoic acid | Human placenta  Human cord blood plasma  Mouse brain |  |
| 11,12-DiHETrE \| 11,12-DiHET | 11,12-Dihydroxyeicosatrienoic acid | Human placenta  Human cord blood plasma  Mouse brain |  |
| 14,15-DiHETrE \| 14,15-DiHET | 14,15-Dihydroxyeicosatrienoic acid | Human placenta  Human cord blood plasma  Mouse brain |  |
| **Arachidonic acid (AA) metabolites obtained through the cyclooxygenase pathway** | | | |
| PGF_2α_ | Prostaglandin F2 alpha | Human placenta  Mouse brain | Pro-inflammatory eicosanoids. Both prostaglandins have acute inflammatory activity. TXB_2_ is a final inactive metabolite from thromboxane A_2_. |
| PGE_2_ | Prostaglandin E2 | Human placenta  Mouse brain |  |
| TXB_2_ | Thromboxane B2 | Human placenta  Human cord blood plasma  Mouse brain |  |
| **Linoleic acid (LA) metabolites obtained through the cytochrome P450 epoxygenase pathway** | | | |
| 9,10-DiHOME | 9,10-Dihydroxy-12Z-octadecenoic acid | Human placenta  Human cord blood plasma  Mouse brain | DiHOMEs and their precursors EpOMEs are toxic mediators. However, 12,13-DiHOME may promote metabolic health. |
| 12,13-DiHOME | 12,13-Dihydroxy-9Z-octadecenoic acid | Human placenta  Human cord blood plasma  Mouse brain |  |
| **Docosahexaenoic acid (DHA) metabolite obtained through the cytochrome P450 epoxygenase pathway** | | | |
| 19,20-DiHDPA | 19,20-Dihydroxydocosapentaenoic acid | Human placenta  Human cord blood plasma  Mouse brain | Stable final metabolite of DHA. |
| **Docosahexaenoic acid (DHA) metabolites obtained through the lipoxygenase pathway** | | | |
| 14-HDHA \| 14-HDoHE | 14-Hydroxydocosahexaenoic acid | Human placenta  Human cord blood plasma  Mouse brain | Anti-inflammatory and beneficial metabolites. |
| 17-HDHA \| 17-HDoHE | 17-Hydroxydocosahexaenoic acid | Human placenta  Mouse brain |  |
| **Eicosapentaenoic acid (EPA) metabolite obtained through the cytochrome P450 ω-hydroxylase pathway** | | | |
| 18-HEPE | 18-Hydroxyeicosapentaenoic acid | Human placenta | Anti-inflammatory and beneficial metabolite. |

Notes: ^1^Main characteristics. See text for more details and references.


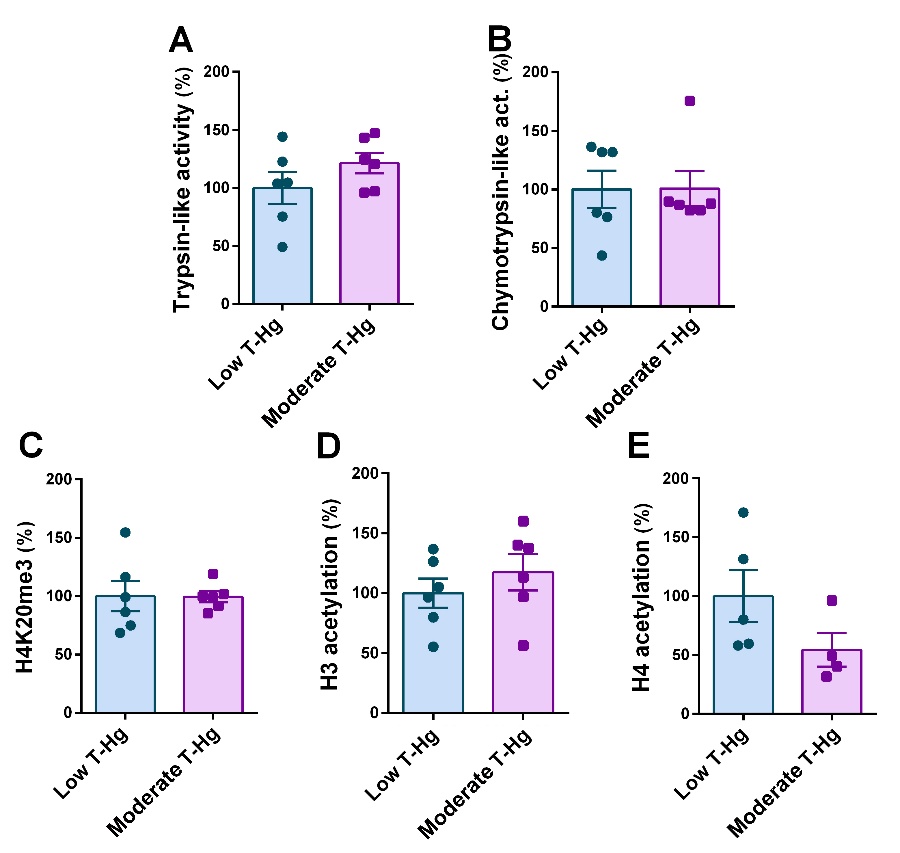


**Supplementary Fig. 1** Proteasome enzymatic activities and histone epigenetic modifications of placental tissue. (A) Trypsin-like activity and (B) Chymotrypsin-like activity of the proteasome (N = 6/group); (C) Levels of trimethylation of lysine 20 on histone H4 (H4K20me3), (C) total H3 acetylation and (E) total H4 acetylation. Results are shown as mean ± SEM (N = 4-6/group). No significant changes associated to prenatal mercury exposure are shown. See Figure 1 of main text for parameters with statistically significant changes. Low T-Hg and moderate T-Hg groups include samples from donors with total mercury levels measured in whole cord blood of 1.4 -6.6 µg/L and 20 - 66 µg/L, respectively.

**Supplementary Table 4** Scores of McCarthy Scales of neurodevelopment at 5 years of age in donors of placental samples.

|  | MSCA^1^ scores | Low T-Hg | | | Moderate T-Hg | | |
| --- | --- | --- | --- | --- | --- | --- | --- |
|  |  | mean | SEM | N | mean | SEM | N |
| MSCA^1^ scores | General cognitive | 81.946 | 8.188 | 4 | 105.306 | 6.692 | 3 |
|  | Verbal | 78.963 | 6.670 | 4 | 112.356 | 4.718 | 3 |
|  | Perceptual performance | 96.595 | 9.593 | 4 | 93.643 | 15.122 | 3 |
|  | Numerical | 80.655 | 10.347 | 4 | 106.040 | 5.831 | 3 |
|  | Memory | 84.283 | 9.574 | 4 | 108.737 | 7.716 | 3 |
|  | Motor | 95.103 | 7.704 | 4 | 105.619 | 7.475 | 3 |
|  | Gross motor | 93.665 | 6.724 | 4 | 110.854 | 3.369 | 3 |
|  | Fine motor | 98.848 | 8.541 | 4 | 97.937 | 11.367 | 3 |
|  | Executive Function | 79.078 | 7.381 | 4 | 102.131 | 3.807 | 3 |
|  | Working Memory | 88.224 | 10.087 | 4 | 104.298 | 3.239 | 3 |

Notes: ^1^MSCA, the McCarthy Scales of Children’s Abilities. No statistical analysis was performed due to the small number of individuals per group, as indicated in the main text. T-Hg, total mercury in cord blood.

**Supplementary Table 5** Spearman correlations between Bayley Scales at 14 months of age and 17 oxylipins detected in fetal and maternal placental tissue pairs for low and moderate mercury exposure groups.

|  |  | Low T-Hg (N = 6) | | | | Moderate T-Hg (N = 6) | | | |
| --- | --- | --- | --- | --- | --- | --- | --- | --- | --- |
|  | BSID^1^ scores | Mental scale | | Psychomotor scale | | Mental scale | | Psychomotor scale | |
|  |  | Spearman  ρ | *p* | Spearman  ρ | *p* | Spearman  ρ | *p* | Spearman  ρ | *p* |
| Oxylipins (nmol/mg) | 5,6-EET | **.943**** | .005 | .143 | .787 | .145 | .784 | .029 | .957 |
|  | 8,9-EET | **.771** | .072 | .143 | .787 | .058 | .913 | -.257 | .623 |
|  | 11,12-EET | **.886*** | .019 | -.029 | .957 | .000 | 1.000 | .086 | .872 |
|  | 14,15-EET | **.771** | .072 | .143 | .787 | -.087 | .870 | -.200 | .704 |
|  | 5,6-DiHET | **.829*** | .042 | .257 | .623 | .551 | .257 | -.600 | .208 |
|  | 8,9-DiHET | .600 | .208 | .429 | .397 | .493 | .321 | -.371 | .468 |
|  | 11,12-DiHET | .116 | .827 | .493 | .321 | .290 | .577 | .086 | .872 |
|  | 14,15-DiHET | .371 | .468 | **.771** | .072 | .290 | .577 | .086 | .872 |
|  | PGF_2α_ | **.771** | .072 | .371 | .468 | .406 | .425 | **-.886*** | .019 |
|  | PGE_2_ | **-.886*** | .019 | .029 | .957 | .058 | .913 | -.543 | .266 |
|  | TXB_2_ | -.486 | .329 | -.143 | .787 | -.058 | .913 | -.657 | .156 |
|  | 9,10-DiHOME | **.943**** | .005 | .143 | .787 | .203 | .700 | -.200 | .704 |
|  | 12,13-DiHOME | .486 | .329 | .143 | .787 | .638 | .173 | .314 | .544 |
|  | 19,20-DiHDPA | .371 | .468 | .086 | .872 | .667 | .148 | -.543 | .266 |
|  | 14-HDoHE | **.886*** | .019 | .143 | .787 | .290 | .577 | **-.829*** | .042 |
|  | 17-HDoHE | **.771** | .072 | .086 | .872 | .232 | .658 | **-.771** | .072 |
|  | 18-HEPE | **.943**** | .005 | -.086 | .872 | .290 | .577 | **-.829*** | .042 |

Notes: ^1^BSID, Bayley Scales of Infant and Toddler Development. Highlighted significant values (* *p* < 0.05, ** *p* < 0.01) and borderline values (*p* < 0.1). T-Hg, total mercury in cord blood. A heat map of these data is shown in the main text (Figure 4A).

**Supplementary Table 6** Spearman correlations between Bayley Scales at 14 months of age and 9 oxylipins detected in cord blood plasma for low and moderate mercury exposure groups.

|  |  | Low T-Hg (N =16) | | | | Moderate T-Hg (N =18) | | | |
| --- | --- | --- | --- | --- | --- | --- | --- | --- | --- |
|  | BSID^1^ scores | Mental scale | | Psychomotor scale | | Mental scale | | Psychomotor scale | |
|  |  | Spearman ρ | *p* | Spearman ρ | *p* | Spearman ρ | *p* | Spearman ρ | *p* |
| Oxylipins (nM) | 5,6-DiHET | .421 | .105 | **.506*** | .046 | .181 | .473 | .103 | .683 |
|  | 8,9-DiHET | **.438** | .090 | .303 | .254 | **.449** | .062 | .127 | .616 |
|  | 11,12-DiHET | .235 | .380 | .326 | .217 | **.515*** | .029 | .025 | .922 |
|  | 14,15-DiHET | .156 | .564 | -.038 | .888 | **.441** | .067 | .042 | .868 |
|  | TXB_2_ | .012 | .966 | **.453** | .078 | **.492*** | .038 | .316 | .202 |
|  | 9,10-DiHOME | -.179 | .506 | .000 | 1.000 | -.028 | .913 | -.368 | .133 |
|  | 12,13-DiHOME | .012 | .966 | .285 | .284 | .001 | .997 | -.261 | .295 |
|  | 19,20-DiHDPA | .418 | .107 | .303 | .254 | .350 | .155 | .305 | .219 |
|  | 14-HDoHE | -.056 | .837 | .221 | .412 | .143 | .570 | .389 | .110 |

Notes: ^1^BSID, Bayley Scales of Infant and Toddler Development. Highlighted significant values (* *p* < 0.05, ** *p* < 0.01) and borderline values (*p* < 0.1). T-Hg, total mercury in cord blood. A heat map of these data is shown in the main text (Figure 4B).

**Supplementary Table 7** Spearman correlations between McCarthy Scales at 5 years of age and 9 oxylipins detected in cord blood plasma for low and moderate mercury exposure groups.

|  |  | Low T-Hg (N = 8) | | | | | | | | | | | | | | | | | | | |
| --- | --- | --- | --- | --- | --- | --- | --- | --- | --- | --- | --- | --- | --- | --- | --- | --- | --- | --- | --- | --- | --- |
|  | MSCA^1^ scores | General cognitive | | Verbal | | Perceptual-performance | | Numerical | | Memory | | Motor | | Gross motor | | Fine motor | | Executive function | | Working memory | |
|  |  | Spearman ρ | *p* | Spearman ρ | *p* | Spearman ρ | *p* | Spearman ρ | *p* | Spearman ρ | *p* | Spearman ρ | *p* | Spearman ρ | *p* | Spearman ρ | *p* | Spearman ρ | *p* | Spearman ρ | *p* |
| Oxylipins (nM) | 5,6-DiHET | .000 | 1.000 | -.167 | .693 | -.571 | .139 | .143 | .736 | -.071 | .867 | -.452 | .260 | .048 | .911 | **-.743*** | .035 | -.263 | .528 | -.143 | .736 |
|  | 8,9-DiHET | .548 | .160 | .381 | .352 | -.476 | .233 | .548 | .160 | .333 | .420 | -.357 | .385 | .143 | .736 | -.479 | .230 | .335 | .417 | .381 | .352 |
|  | 11,12-DiHET | .024 | .955 | .190 | .651 | **-.762*** | .028 | -.024 | .955 | -.048 | .911 | .024 | .955 | .190 | .651 | -.335 | .417 | -.096 | .821 | -.048 | .911 |
|  | 14,15-DiHET | -.476 | .233 | .262 | .531 | -.452 | .260 | -.619 | .102 | -.143 | .736 | .214 | .610 | .119 | .779 | .072 | .866 | -.359 | .382 | -.286 | .493 |
|  | TXB_2_ | .167 | .693 | -.095 | .823 | .262 | .531 | .095 | .823 | -.381 | .352 | -.167 | .693 | .119 | .779 | -.299 | .471 | .084 | .844 | -.214 | .610 |
|  | 9,10-DiHOME | .119 | .779 | .500 | .207 | -.048 | .911 | -.071 | .867 | -.024 | .955 | .286 | .493 | .286 | .493 | .287 | .490 | .335 | .417 | .143 | .736 |
|  | 12,13-DiHOME | .214 | .610 | **.643** | .086 | .214 | .610 | -.190 | .651 | -.143 | .736 | .548 | .160 | .286 | .493 | .539 | .168 | .443 | .272 | .071 | .867 |
|  | 19,20-DiHDPA | .071 | .867 | .119 | .779 | .000 | 1.000 | -.095 | .823 | -.048 | .911 | .238 | .570 | -.190 | .651 | .263 | .528 | .072 | .866 | .024 | .955 |
|  | 14-HDoHE | -.143 | .736 | .143 | .736 | .024 | .955 | -.524 | .183 | **-.690** | .058 | .333 | .420 | .548 | .160 | -.252 | .548 | -.252 | .548 | **-.714*** | .047 |
|  |  | **Moderate T-Hg (N = 17)** | | | | | | | | | | | | | | | | | | | |
| Oxylipins (nM) | 5,6-DiHET | .108 | .680 | -.087 | .740 | -.007 | .978 | .270 | .295 | .255 | .323 | -.157 | .548 | .010 | .970 | -.245 | .343 | .012 | .963 | .154 | .554 |
|  | 8,9-DiHET | .223 | .390 | .186 | .474 | .005 | .985 | .338 | .184 | .306 | .232 | -.142 | .586 | .037 | .889 | -.123 | .639 | .240 | .353 | .277 | .282 |
|  | 11,12-DiHET | .360 | .155 | .217 | .403 | .118 | .653 | **.446** | .073 | .203 | .434 | -.113 | .667 | -.152 | .560 | .054 | .837 | .341 | .181 | **.515*** | .035 |
|  | 14,15-DiHET | .191 | .462 | .055 | .833 | -.044 | .866 | **.453** | .068 | -.042 | .874 | -.230 | .374 | -.184 | .480 | -.100 | .701 | .250 | .333 | **.608**** | .010 |
|  | TXB_2_ | **.434** | .082 | .037 | .889 | **.426** | .088 | **.534*** | .027 | **.525*** | .031 | .370 | .144 | .125 | .633 | .341 | .181 | .275 | .286 | **.444** | .074 |
|  | 9,10-DiHOME | -.098 | .708 | -.224 | .387 | .110 | .673 | -.147 | .573 | -.017 | .948 | .071 | .786 | -.025 | .926 | .108 | .680 | -.233 | .368 | -.206 | .428 |
|  | 12,13-DiHOME | -.105 | .687 | -.335 | .189 | .181 | .486 | -.142 | .586 | -.010 | .970 | .186 | .474 | .113 | .667 | .154 | .554 | -.316 | .216 | -.206 | .428 |
|  | 19,20-DiHDPA | -.279 | .277 | -.103 | .694 | -.407 | .105 | .181 | .486 | .034 | .896 | -.292 | .256 | .056 | .830 | -.412 | .101 | -.275 | .286 | .147 | .573 |
|  | 14-HDoHE | -.346 | .174 | -.327 | .200 | -.184 | .480 | .037 | .889 | .172 | .510 | -.012 | .963 | .282 | .273 | -.240 | .353 | -.328 | .198 | -.069 | .794 |

Notes: ^1^MSCA, McCarthy Scales of Children’s Abilities. Highlighted significant values (* *p* < 0.05, ** *p* < 0.01) and borderline values (*p* < 0.1). T-Hg, total mercury in cord blood. A heat map of these data is shown in the main text (Figure 4C).

**Supplementary Table 8** Spearman correlations between McCarthy Scales at 5 years of age and 17 oxylipins detected in fetal and maternal placental tissue pairs for all children.

|  | All children (N = 7) | | | | | | | | | | | | | | | | | | | | |
| --- | --- | --- | --- | --- | --- | --- | --- | --- | --- | --- | --- | --- | --- | --- | --- | --- | --- | --- | --- | --- | --- |
|  | MSCA^1^ scores | General cognitive | | Verbal | | Perceptual-performance | | Numerical | | Memory | | Motor | | Gross motor | | Fine motor | | Executive function | | Working memory | |
|  |  | Spearman ρ | *p* | Spearman ρ | *p* | Spearman ρ | *p* | Spearman ρ | *p* | Spearman ρ | *p* | Spearman ρ | *p* | Spearman ρ | *p* | Spearman ρ | *p* | Spearman ρ | *p* | Spearman ρ | *p* |
| Oxylipins (nmol/mg) | 5,6-EET | -.071 | .879 | -.071 | .879 | .250 | .589 | -.429 | .337 | -.536 | .215 | .357 | .432 | -.286 | .535 | .286 | .535 | -.071 | .879 | -.643 | .119 |
|  | 8,9-EET | .214 | .645 | .179 | .702 | .214 | .645 | -.107 | .819 | -.214 | .645 | .464 | .294 | -.036 | .939 | .286 | .535 | .214 | .645 | -.429 | .337 |
|  | 11,12-EET | .179 | .702 | .143 | .760 | .429 | .337 | -.107 | .819 | -.214 | .645 | .607 | .148 | -.071 | .879 | .536 | .215 | .179 | .702 | -.429 | .337 |
|  | 14,15-EET | .071 | .879 | .036 | .939 | .250 | .589 | -.179 | .702 | -.250 | .589 | .429 | .337 | -.107 | .819 | .357 | .432 | .071 | .879 | -.464 | .294 |
|  | 5,6-DiHET | .571 | .180 | .643 | .119 | .571 | .180 | .143 | .760 | -.036 | .939 | **.929**** | .003 | .393 | .383 | **.679** | .094 | .571 | .180 | -.214 | .645 |
|  | 8,9-DiHET | **.679** | .094 | **.750** | .052 | -.143 | .760 | .357 | .432 | .071 | .879 | .357 | .432 | .607 | .148 | -.179 | .702 | **.679** | .094 | .000 | 1.000 |
|  | 11,12-DiHET | .321 | .482 | .321 | .482 | -.357 | .432 | .357 | .432 | .179 | .702 | -.214 | .645 | .286 | .535 | -.500 | .253 | .321 | .482 | .286 | .535 |
|  | 14,15-DiHET | .179 | .702 | .250 | .589 | -.643 | .119 | .071 | .879 | -.179 | .702 | -.286 | .535 | .357 | .432 | **-.714** | .071 | .179 | .702 | -.143 | .760 |
|  | PGF_2α_ | .107 | .819 | .036 | .939 | .536 | .215 | -.143 | .760 | -.179 | .702 | .571 | .180 | -.250 | .589 | .607 | .148 | .107 | .819 | -.357 | .432 |
|  | PGE_2_ | .464 | .294 | .214 | .645 | .429 | .337 | .500 | .253 | .571 | .180 | .214 | .645 | -.321 | .482 | .250 | .589 | .464 | .294 | .536 | .215 |
|  | TXB_2_ | -.429 | .337 | -.536 | .215 | .107 | .819 | -.357 | .432 | -.179 | .702 | -.143 | .760 | -.571 | .180 | .143 | .760 | -.429 | .337 | -.250 | .589 |
|  | 9,10-DiHOME | .500 | .253 | .571 | .180 | .036 | .939 | .179 | .702 | -.179 | .702 | .500 | .253 | .464 | .294 | .071 | .879 | .500 | .253 | -.286 | .535 |
|  | 12,13-DiHOME | .643 | .119 | **.714** | .071 | .036 | .939 | .464 | .294 | .286 | .535 | .321 | .482 | .571 | .180 | -.036 | .939 | .643 | .119 | .321 | .482 |
|  | 19,20-DiHDPA | .179 | .702 | .179 | .702 | .464 | .294 | .143 | .760 | .286 | .535 | .214 | .645 | -.071 | .879 | .357 | .432 | .179 | .702 | .429 | .337 |
|  | 14-HDoHE | .286 | .535 | .429 | .337 | .464 | .294 | .000 | 1.000 | .000 | 1.000 | **.750** | .052 | .464 | .294 | **.679** | .094 | .286 | .535 | -.179 | .702 |
|  | 17-HDoHE | .179 | .702 | .286 | .535 | **.679** | .094 | -.036 | .939 | .036 | .939 | **.714** | .071 | .214 | .645 | **.821*** | .023 | .179 | .702 | -.036 | .939 |
|  | 18-HEPE | .536 | .215 | .571 | .180 | .643 | .119 | .214 | .645 | .107 | .819 | **.929**** | .003 | .357 | .432 | **.786*** | .036 | .536 | .215 | -.107 | .819 |

Notes: ^1^MSCA, McCarthy Scales of Children’s Abilities. Highlighted significant values (* *p* < 0.05, ** *p* < 0.01) and borderline values (*p* < 0.1). Correlations for either low or moderate mercury exposure groups were not analyzed because of the low number of data per group (N = 4 and N = 3, respectively).

**Supplementary Table 9A** Moderate association between three selected McCarthy Scales at 5 years of age and 9 oxylipins detected in cord blood plasma for all children (N = 25).

|  | MSCA^1^ scores | General cognitive | | | | | Gross motor | | | | | Executive function | | | | |
| --- | --- | --- | --- | --- | --- | --- | --- | --- | --- | --- | --- | --- | --- | --- | --- | --- |
|  |  | Multivariate linear  regression | | | Spearman correlation | | Multivariate linear  regression | | | Spearman correlation | | Multivariate linear  regression | | | Spearman correlation | |
|  |  | Beta | 95% CI | *p* | Rho | *p* | Beta | 95% CI | *p* | Rho | *p* | Beta | 95% CI | *p* | Rho | *p* |
| Oxylipins (nM) | 5,6-DiHET | -5.748 | -21.8;  10.3 | 0.463 | 0.077 | 0.716 | 3.703 | -12.5;  19.9 | 0.638 | .071 | 0.737 | -4.258 | -20.9  12.4 | 0.600 | -0.005 | 0.983 |
|  | 8,9-DiHET | 3.234 | -4.05;  10.5 | 0.366 | 0.258 | 0.212 | 3.255 | -3.14;  9.65 | 0.300 | 0.138 | 0.512 | 4.321 | -3.11  11.8 | 0.239 | 0.234 | 0.260 |
|  | 11,12-DiHET | 3.142 | -2.63;  8.91 | 0.270 | 0.228 | 0.272 | 2.552 | -2.55;  7.66 | 0.308 | -0.056 | 0.790 | 4.657 | -1.11  10.4 | 0.108 | 0.296 | 0.151 |
|  | 14,15-DiHET | 0.964 | -4.92;  6.85 | 0.736 | -0.030 | 0.887 | 2.662 | -2.38;  7.70 | 0.283 | -0.142 | 0.497 | 2.584 | -3.40  8.57 | 0.379 | 0.106 | 0.615 |
|  | TXB_2_ | 0.300 | -0.50;  1.10 | 0.445 | **0.413^*^** | 0.040 | 0.054 | -0.67;  0.77 | 0.878 | 0.182 | 0.383 | -0.148 | -0.99  0.69 | 0.717 | 0.197 | 0.346 |
|  | 9,10-DiHOME | -0.033 | -2.02;  1.96 | 0.972 | -0.026 | 0.901 | 0.201 | -1.55;  1.95 | 0.813 | 0.152 | 0.467 | -0.039 | -2.10  2.02 | 0.969 | -0.168 | 0.421 |
|  | 12,13-DiHOME | -0.256 | -3.08;  2.57 | 0.852 | 0.010 | 0.962 | 0.176 | -2.32;  2.67 | 0.884 | 0.198 | 0.344 | -0.431 | -3.35  2.49 | 0.761 | -0.209 | 0.317 |
|  | 19,20-DiHDPA | -1.976 | -6.12;  2.16 | 0.331 | -0.161 | 0.443 | 0.282 | -3.47;  4.03 | 0.876 | -0.113 | 0.590 | -1.558 | -5.89  2.77 | 0.462 | -0.235 | 0.259 |
|  | 14-HDoHE | -0.202 | -1.66;  1.25 | 0.775 | -0.285 | 0.167 | 0.853 | -0.51;  2.22 | 0.206 | **0.396^*^** | 0.050 | -0.534 | -2.02  0.95 | 0.463 | **-0.366** | 0.072 |

Notes: ^1^MSCA, McCarthy Scales of Children’s Abilities. Highlighted significant values (* *p* < 0.05) and borderline values (*p* < 0.1). The two statistical models were independently calculated with the same data. Multivariate linear regression model adjusted for cord blood T-Hg concentration, maternal seafood intake and newborn sex. The four MSCA scales showed some significant associations in the Spearman correlation model. Association analyses of other MSCA scales are shown in Table 4 and Supplementary Table 9B.

**Supplementary Table 9B** Lack of association between three selected McCarthy Scales at 5 years of age and 9 oxylipins detected in cord blood plasma for all children (N = 25).

|  | MSCA^1^ scores | Verbal | | | | | Memory | | | | | Motor | | | | |
| --- | --- | --- | --- | --- | --- | --- | --- | --- | --- | --- | --- | --- | --- | --- | --- | --- |
|  |  | Multivariate linear  regression | | | Spearman correlation | | Multivariate linear  regression | | | Spearman correlation | | Multivariate linear  regression | | | Spearman correlation | |
|  |  | Beta | 95% CI | *p* | Rho | *p* | Beta | 95% CI | *p* | Rho | *p* | Beta | 95% CI | *p* | Rho | *p* |
| Oxylipins (nM) | 5,6-DiHET | -7.519 | -24.7;  9.65 | 0.372 | -0.055 | 0.795 | -1.446 | -16.0;  13.08 | 0.838 | 0.166 | 0.428 | -6.084 | -22.4;  10.21 | 0.445 | -0.227 | 0.275 |
|  | 8,9-DiHET | 3.647 | -4.20;  11.50 | 0.344 | 0.244 | 0.240 | 3.660 | -2.78;  10.10 | 0.250 | 0.281 | 0.174 | -1.874 | -9.40;  5.66 | 0.609 | -0.133 | 0.526 |
|  | 11,12-DiHET | 3.551 | -2.66;  9.76 | 0.247 | 0.242 | 0.245 | 1.404 | -3.89;  6.70 | 0.586 | 0.081 | 0.701 | -0.137 | -6.20;  5.93 | 0.963 | -0.098 | 0.642 |
|  | 14,15-DiHET | 1.244 | -5.10;  7.59 | 0.687 | 0.088 | 0.675 | -0.471 | -5.75;  4.81 | 0.854 | -0.080 | 0.704 | 0.298 | -5.71;  6.31 | 0.919 | -0.166 | 0.427 |
|  | TXB_2_ | -0.392 | -1.25;  0.47 | 0.353 | -0.019 | 0.929 | 0.281 | -0.44;  1.00 | 0.423 | 0.188 | 0.369 | 0.590 | -0.19;  1.37 | 0.132 | 0.306 | 0.137 |
|  | 9,10-DiHOME | -0.441 | -2.58;  1.70 | 0.672 | -0.039 | 0.854 | 0.330 | -1.45;  2.11 | 0.703 | -0.042 | 0.841 | 0.247 | -1.78;  2.27 | 0.802 | 0.138 | 0.512 |
|  | 12,13-DiHOME | -1.102 | -4.11;  1.91 | 0.454 | -0.106 | 0.613 | 0.358 | -2.17;  2.89 | 0.771 | 0.002 | 0.994 | 0.789 | -2.07;  3.65 | 0.572 | 0.288 | 0.163 |
|  | 19,20-DiHDPA | -2.869 | -7.25;  1.51 | 0.187 | -0.028 | 0.895 | -0.453 | -4.25;  3.34 | 0.806 | 0.074 | 0.726 | -1.045 | -5.34;  3.25 | 0.617 | -0.246 | 0.236 |
|  | 14-HDoHE | -0.848 | -2.37;  0.68 | 0.259 | -0.218 | 0.295 | 0.114 | -1.19;  1.42 | 0.857 | -0.062 | 0.770 | 1.023 | -0.38;  2.43 | 0.145 | 0.080 | 0.704 |

Notes: ^1^MSCA, McCarthy Scales of Children’s Abilities. The two statistical models were independently calculated with the same data. Multivariate linear regression model adjusted for cord blood T-Hg concentration, maternal seafood intake and newborn sex. The three MSCA scales displayed showed no significant association in any of both models. Association analyses of other MSCA scales are shown in Table 4 and Supplementary Table 9A

**
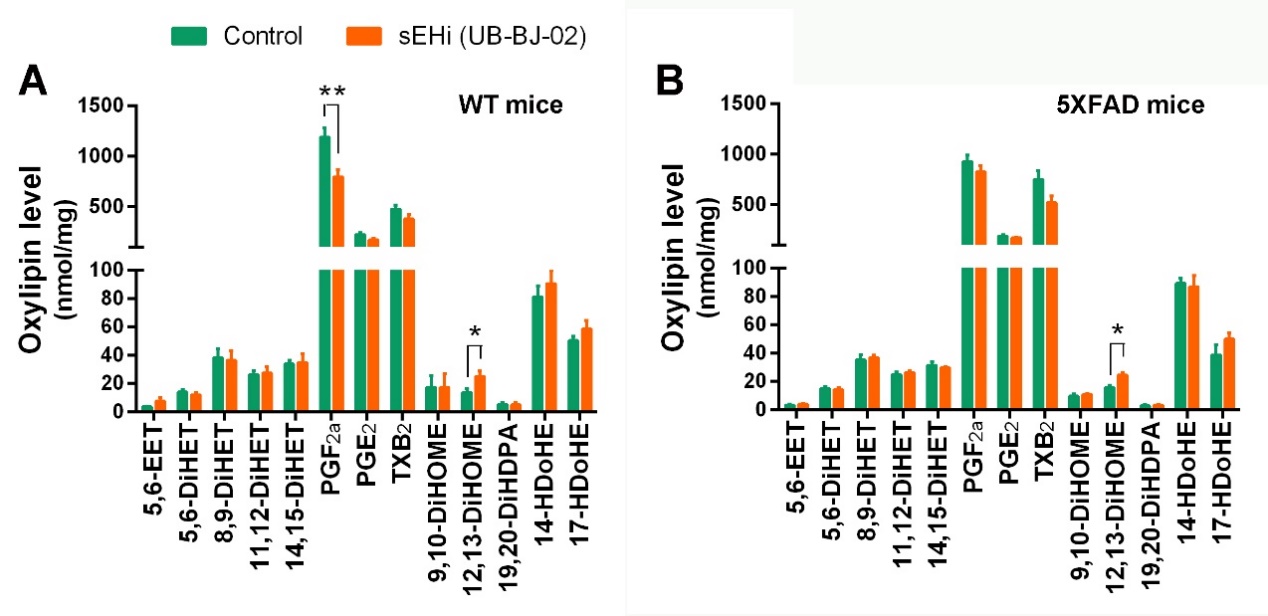
**

**Supplementary Fig. 2** Oxylipin profile in cerebral cortical tissue of adult WT mice (A) and 5XFAD mice (B) after chronic treatment with the sEHi UB-BJ-02 or vehicle. UPLC-MS/MS analysis showed detectable levels of 13 oxylipins. Result are shown as mean ± SEM (N = 5 per group). Changes in oxylipin levels within each strain were analyzed by Student’s *t* test: * *p* < 0.05, ** *p* < 0.01. The results of the ANOVA analysis for pooled samples of both strains are shown in the main text (Figure 5).

**Supplementary Table 10** Spearman correlations between prenatal exposure to cadmium, lead and mercury exposure and 9 oxylipins detected in cord blood plasma for all children.

|  |  | Cd (N = 34) | | Pb (N = 34) | | T-Hg (N = 39) | |
| --- | --- | --- | --- | --- | --- | --- | --- |
|  |  | Spearman correlation | | Spearman correlation | | Spearman correlation | |
|  |  | Rho | *p* | Rho | *p* | Rho | *p* |
| Oxylipins (nM) | 5,6-DiHET | -0.173 | 0.329 | -0.039 | 0.827 | 0.153 | 0.351 |
|  | 8,9-DiHET | -0.082 | 0.643 | -0.019 | 0.915 | 0.150 | 0.362 |
|  | 11,12-DiHET | -0.084 | 0.638 | 0.150 | 0.398 | 0.127 | 0.440 |
|  | 14,15-DiHET | -0.047 | 0.791 | 0.074 | 0.678 | -0.030 | 0.854 |
|  | TXB_2_ | 0.011 | 0.949 | 0.077 | 0.666 | -0.221 | 0.176 |
|  | 9,10-DiHOME | 0.227 | 0.197 | 0.071 | 0.690 | -0.035 | 0.831 |
|  | 12,13-DiHOME | 0.147 | 0.406 | -0.054 | 0.762 | 0.089 | 0.589 |
|  | 19,20-DiHDPA | -0.211 | 0.232 | -0.143 | 0.419 | **0.470*** | 0.003 |
|  | 14-HDoHE | -0.158 | 0.374 | 0.086 | 0.629 | 0.092 | 0.579 |

Notes: UPLC-MS/MS analysis of 9 detected oxylipins. Cadmium (Cd) and lead (Pb) were measure in maternal urine, and total mercury (T-Hg) in whole cord blood. Statistics * *p* < 0.01.

**Supplementary Table 11A**  Association between selected neurodevelopmental scales and 9 oxylipins detected in cord blood plasma for all children using multivariate linear regression models, additionally adjusted for prenatal exposures to cadmium and lead.

|  |  | BSID^1^ scores (N = 34) | | | MSCA^2^ scores (N = 25) | | | | | | | | | | | | | | | | | |
| --- | --- | --- | --- | --- | --- | --- | --- | --- | --- | --- | --- | --- | --- | --- | --- | --- | --- | --- | --- | --- | --- | --- |
|  |  | Mental scale | | | Perceptual-performance | | | | Numerical | | | Fine motor | | | Working memory | | | | Executive function | | | |
|  |  | Beta | 95%  CI | *p* | | Beta | 95%  CI | *p* | Beta | 95%  CI | *p* | Beta | 95%  CI | *p* | Beta | 95%  CI | *p* | Beta- | | 95%  CI | *p* |  |
| Oxylipins (nM) | 5,6-DiHET | 14,82 | -6.39; 36.04 | 0,163 | | -7.29 | -28.03; 13.45 | 0.470 | 8.66 | -8.25;  25.57 | 0.296 | -15.69 | -35.65;  4.28 | 0.116 | 2.54 | -13.17:  18.25 | 0.738 | 0.648 | | -15.62;  16.92 | 0.934 |  |
|  | 8,9-DiHET | **10.99**** | 3.38;  18.61 | 0.006 | | -2.30 | -11.51;  6.90 | 0.605 | **6.77** | -0.15;  13.69 | 0.055 | -5.85 | -14.83;  3.14 | 0.189 | 4.35 | -2.25;  10.96 | 0.183 | 4.56 | | -2.25;  11.36 | 0.177 |  |
|  | 11,12-DiHET | **7.03*** | 1.76;12.31 | 0.011 | | -1.05 | -8.46;  6.36 | 0.770 | **5.41** | -0.13;  10.95 | 0.055 | -2.38 | -9.84;  5.09 | 0.512 | **4.90** | -0.10;  9.90 | 0.054 | **4.73** | | -0.53;  9.97 | 0.074 |  |
|  | 14,15-DiHET | 4.64 | -0.98;  10.26 | 0.101 | | -1.21 | -8.58;  6.17 | 0.735 | 2.96 | -2.99;  8.92 | 0.309 | -1.907 | -9.38;  5.56 | 0.598 | 3.96 | -1.22;  9.14 | 0.125 | 2.33 | | -3.27;  7.93 | 0.394 |  |
|  | TXB_2_ | -0.298 | -1.06;  0.46 | 0.429 | | **1.01*** | 0.11;  1.90 | 0.029 | 0.363 | -0.46;  1.19 | 0.368 | **0.866** | -0.08;  1.81 | 0.071 | 0.012 | -0.75;  0.78 | 0.975 | -0.056 | | -0.85;  0.73 | 0.882 |  |
|  | 9,10-DiHOME | -0.604 | -3.09;  1.88 | 0.621 | | 0.22 | -2.31;  2.75 | 0.850 | -0.065 | -2.16;  2.03 | 0.948 | -0.181 | -2.75;  2.39 | 0.884 | -0.188 | -2.08;  1.70 | 0.837 | -0.357 | | -2.30;  1.59 | 0.705 |  |
|  | 12,13-DiHOME | -0.656 | -4.29; 2.98 | 0.713 | | 0.581 | -3.05;  4.21 | 0.740 | -0.339 | -3.35;  2.67 | 0.816 | 0.355 | -3.35;  4.06 | 0.842 | -0.411 | -3.13;  2.31 | 0.755 | -0.942 | | -3.71;  1.83 | 0.485 |  |
|  | 19,20-DiHDPA | **5.83*** | 0.87;  10.79 | 0.023 | | -2.30 | -7.61;  3.01 | 0.375 | 2.10 | -2.28:  6.47 | 0.328 | -1.816 | -7.27;  3.64 | 0.493 | 2.17 | -1.75;  6.09 | 0.259 | -1.10 | | -5.26,  3.06 | 0.585 |  |
|  | 14-HDoHE | -0.812 | -2.47;  0.85 | 0.324 | | 0.623 | -1.23;  2.48 | 0.489 | 0.368 | -1.18;  1.92 | 0.623 | 0.426 | -1.48;  2.33 | 0.644 | -0.003 | -1,41;  1.41 | 0.996 | -0.192 | | -1.64;  1.26 | 0.784 |  |

Notes: ^1^BSID, Bayley Scales of Infant and Toddler Development. ^2^MSCA, McCarthy Scales of Children’s Abilities. Highlighted significant values (* *p* < 0.05, ** *p* < 0.01) and borderline values (*p* < 0.1). Multivariate linear regression models adjusted for cord blood T-Hg concentration, maternal seafood consumption, newborn sex, and additionally adjusted for cadmium and lead measured in maternal urine samples during pregnancy. Results for other BSID and MSCA scales without significant association are shown in Supplementary Table 11B. Comparison with the multivariate linear regression associations shown in the Tables 3-4 of the main text indicates minor changes after adjustment here for cadmium and lead, as follows: borderline significance was lost for the isomer 5,6-DiHET with the Bayley mental scale and gained for 11,12-DiEHT with the McCarthy executive function scale; in the McCarthy fine motor scale, TXB2 gained borderline significance, while the borderline negative association with 5,6-DiHET disappeared.

**Supplementary Table 11B** Lack of association between six selected neurodevelopmental scales and 9 oxylipins detected in cord blood plasma for all children using multivariate linear regression models, additionally adjusted for prenatal exposures to cadmium and lead.

|  |  | BSID^1^ scores (N = 34) | | | MSCA^1^ scores (N = 25) | | | | | | | | | | | | | | | |
| --- | --- | --- | --- | --- | --- | --- | --- | --- | --- | --- | --- | --- | --- | --- | --- | --- | --- | --- | --- | --- |
|  |  | Psychomotor scale | | | General cognitive | | | | Gross motor | | | Verbal | | | Memory | | | Motor | | |
|  |  | Beta | 95%  CI | *p* | | Beta | 95%  CI | *p* | Beta | 95%  CI | *p* | Beta | 95% CI | *p* | Beta | 95% CI | *p* | Beta | 95% CI | *p* |
| Oxylipins (nM) | 5,6-DiHET | 8.59 | -10.71;  27.80 | 0.368 | | -1.48 | -17.28;  14.32 | 0.846 | 4.88 | -9.41;  19.16 | 0.483 | -2.67 | -19.50;  14.16 | 0.743 | 2.16 | -12.17;  16.50 | 0.755 | -7.54 | -24.36;  9.29 | 0.359 |
|  | 8,9-DiHET | 3.74 | -3.98;  11.46 | 0.328 | | 3.54 | -3.20;  10.29 | 0.285 | 3.86 | -2.23;  9.95 | 0.200 | 3.94 | -3.25;  11.12 | 0.265 | 3.99 | -2.03;  10.01 | 0.181 | -1.51 | --9.07;  6.05 | 0.679 |
|  | 11,12-DiHET | 2.20 | -3.07;  7.47 | 0.398 | | 3.28 | -2.06;  8.62 | 0.213 | 2.81 | -2.11;  7.72 | 0.246 | 3.65 | -2.02;  9.33 | 0.193 | **1.58** | -3.44;  6.59 | 0.517 | 0.204 | -5.88;  6.28 | 0.944 |
|  | 14,15-DiHET | 0.513 | -4.76;  5.78 | 0.843 | | 0.823 | -4.72;  6.37 | 0.759 | 3.31 | -1.52;  8.13 | 0.167 | 1.01 | -4.91;  6.92 | 0.725 | -0.505 | -5.55;  4.54 | 0.836 | 0.840 | -5.20,  6.88 | 0.774 |
|  | TXB_2_ | -0.072 | -0.76;  0.61 | 0.830 | | 0.400 | -0.34;  1.14 | 0.271 | 0.044 | -0.66;  0.75 | 0.897 | -0.295 | -1.10;  0.51 | 0.453 | -0.370 | -0.30;  1.04 | 0.263 | 0.629 | -0,15;  1,40 | 0.106 |
|  | 9,10-DiHOME | -0.163 | -2.34;  2.95 | 0.881 | | -0.385 | -2.28;  1.51 | 0.674 | 0.273 | -1.46;  2.01 | 0.745 | -0.822 | -2.81;  1.17 | 0.397 | 0.015 | -1.71;  1.74 | 0.986 | 0.040 | -2.03;  2.11 | 0.968 |
|  | 12,13-DiHOME | 0.435 | -2.80;  3.67 | 0.784 | | -0.834 | -3.54;  1.87 | 0.525 | 0.325 | -2.18;  2.83 | 0.788 | -1.74 | -4.53;  1.05 | 0.206 | -1.05 | -8.46;  6.36 | 0.770 | 0.400 | -2,58;  3.37 | 0.781 |
|  | 19,20-DiHDPA | 3.66 | -1.00;  8.32 | 0.119 | | -1.74 | -6.73;  2.24 | 0.370 | -0.359 | -4.09;  3.37 | 0.842 | -2.54 | -6.71;  1.63 | 0.217 | -0.329 | -4.03;  -3.38 | 0.854 | -1.82 | -6.17;  2.53 | 0.392 |
|  | 14-HDoHE | 0.045 | -1,46;  1.55 | 0.952 | | 0.102 | -1.31; 1.51 | 0.881 | 0.841 | -0.38;  2.07 | 0.167 | -5.526 | -2.01;  0.096 | 0.466 | 0.350 | -0.92;  1.62 | 0.571 | 0.921 | -0.55; 2.39 | 0.205 |

Notes: ^1^BSID, Bayley Scales of Infant and Toddler Development. ^2^MSCA, McCarthy Scales of Children’s Abilities. Multivariate linear regress ion models adjusted for cord blood T-Hg concentration, maternal seafood consumption, newborn sex, and additionally adjusted for cadmium and lead measured in maternal urine samples during pregnancy. Association analysis of other BSID and MSCA scales showing significant association are shown in Table 6.

**Supplementary Table 12** Mean oxylipin levels in cord blood plasma of female and male groups.

|  |  | Low T-Hg | | | | | | Moderate T-Hg | | | | | |
| --- | --- | --- | --- | --- | --- | --- | --- | --- | --- | --- | --- | --- | --- |
|  |  | Females | | | Males | | | Females | | | Males | | |
|  |  | mean | SEM | N | mean | SEM | N | mean | SEM | N | mean | SEM | N |
| Oxylipins (nM) | 5,6-DiHET | 0.448 | 0.046 | 10 | 0.766 | 0.204 | 10 | 0.601 | 0.064 | 5 | 0.546 | 0.093 | 14 |
|  | 8,9-DiHET | 2.707 | 0.208 | 10 | 3.597 | 0.464 | 10 | 3.522 | 0.127 | 5 | 2.924 | 0.222 | 14 |
|  | 11,12-DiHET | 3.317 | 0.382 | 10 | 3.377 | 0.283 | 10 | 4.297 | 0.457 | 5 | 3.101 | 0.288 | 14 |
|  | 14,15-DiHET | 4.395 | 0.331 | 10 | 4.356 | 0.401 | 10 | 5.127 | 0.636 | 5 | 4.029 | 0.306 | 14 |
|  | TXB_2_ | 3.531 | 1.271 | 10 | 6.401 | 3.392 | 10 | 7.950 | 5.612 | 5 | 2.957 | 1.247 | 14 |
|  | 9,10-DiHOME | 2.654 | 0.320 | 10 | 4.478 | 1.375 | 10 | 3.839 | 1.252 | 5 | 4.078 | 0.889 | 14 |
|  | 12,13-DiHOME | 2.534 | 0.232 | 10 | 3.307 | 0.884 | 10 | 3.038 | 0.852 | 5 | 3.582 | 0.632 | 14 |
|  | 19,20-DiHDPA | 2.832 | 0.259 | 10 | 2.249 | 0.151 | 10 | 3.328 | 0.323 | 5 | 3.785 | 0.430 | 14 |
|  | 14-HDoHE | 1.135 | 0.343 | 10 | 2.586 | 1.245 | 10 | 2.046 | 0.879 | 5 | 3.115 | 1.290 | 14 |

Notes: UPLC-MS/MS analysis of 9 detected oxylipins. Three oxylipins showed sex related changes (8,9-DiHET, 11,12-DiHET and 14,15-DiHET). Preliminary statistics: Two-way ANOVA showed a significant effect of sex on the level of oxylipin 14,15-DiHET (*p* = 0.047) and a significant interaction between sex and mercury exposure for the levels of 8,9-DiHET (*p* = 0.007) and 11,12-DiHET (*p* = 0.047).

**Supplementary Table 13** Mean McCarthy scores in female and male children at 5 years, from whom cord blood samples were collected.

|  |  | Low T-Hg | | | | | | Moderate T-Hg | | | | | |
| --- | --- | --- | --- | --- | --- | --- | --- | --- | --- | --- | --- | --- | --- |
|  |  | Females | | | Males | | | Females | | | Males | | |
|  |  | mean | SEM | N | mean | SEM | N | mean | SEM | N | mean | SEM | N |
| MSCA^1^ scores | General cognitive | 91.977 | 4.172 | 5 | 104.422 | 6.003 | 3 | 104.662 | 5.216 | 5 | 94.911 | 3.190 | 12 |
|  | Verbal | 93.550 | 4.318 | 5 | 108.288 | 6.823 | 3 | 106.092 | 5.472 | 5 | 96.864 | 3.790 | 12 |
|  | Perceptual performance | 95.464 | 5.980 | 5 | 90.165 | 7.248 | 3 | 102.686 | 6.267 | 5 | 95.017 | 4.671 | 12 |
|  | Numerical | 93.560 | 4.934 | 5 | 111.285 | 6.521 | 3 | 100.801 | 4.068 | 5 | 96.368 | 3.553 | 12 |
|  | Memory | 91.668 | 3.246 | 5 | 101.890 | 8.755 | 3 | 102.522 | 5.203 | 5 | 96.919 | 2.741 | 12 |
|  | Motor | 100.168 | 6.618 | 5 | 100.194 | 0.356 | 3 | 95.792 | 4.288 | 5 | 101.591 | 4.204 | 12 |
|  | Gross motor | 99.104 | 6.415 | 5 | 112.950 | 3.597 | 3 | 89.023 | 2.966 | 5 | 105.747 | 3.460 | 12 |
|  | Fine motor | 101.445 | 7.019 | 5 | 86.806 | 4.246 | 3 | 106.040 | 6.163 | 5 | 96.431 | 5.034 | 12 |
|  | Executive Function | 95.237 | 5.727 | 5 | 104.859 | 7.790 | 3 | 102.873 | 6.122 | 5 | 92.960 | 2.968 | 12 |
|  | Working Memory | 101.250 | 3.693 | 5 | 107.397 | 5.916 | 3 | 101.427 | 4.161 | 5 | 96.522 | 3.631 | 12 |

Notes: ^1^MSCA, the McCarthy Scales of Children’s Abilities. Four MSCA scores showed sex related changes (general cognitive, verbal, numerical and gross motor). Preliminary statistics: Two-way ANOVA showed a significant effect of sex on the gross motor score (*p* = 0.007) and a significant interaction between sex and mercury exposure for scores in general cognitive (p = 0.034), verbal (p = 0.042), and numerical (*p* = 0.044) domains.
